# Patient satisfaction among national health insurance enrollees in an accredited hospital of Kathmandu Valley: A cross-sectional, mixed methods study

**DOI:** 10.1371/journal.pone.0345353

**Published:** 2026-03-20

**Authors:** Stuti Shrestha, Sudip Khanal, Aashish Acharya, Anisha Chalise

**Affiliations:** 1 Department of Public Health, Manmohan Memorial Institute of Health Sciences, Kathmandu, Bagmati Province, Nepal; 2 Center for Research on Environment, Health and Population Activities, Nepal; Newcastle University, UNITED KINGDOM OF GREAT BRITAIN AND NORTHERN IRELAND

## Abstract

**Background:**

The national health insurance program was piloted in 2016 in Nepal with a political commitment of reducing out-of-pocket expenditure and improving access to quality healthcare services. However, it has been facing challenges of declining renewal rates and high drop-outs over the years. The study sought to assess patient satisfaction and factors influencing satisfaction among national health insurance enrollees.

**Method:**

A sequential explanatory mixed methods study was conducted in a provincial government hospital of Nepal. Patient satisfaction was first assessed among 391 insured patients, followed by in-depth interviews with seven healthcare providers to explore barriers to healthcare provision underlying the observed levels of patient satisfaction in quantitative survey. Both insured patients and providers were purposively selected. Quantitative data was analyzed using descriptive and bivariate analysis, as well as ordinal logistic regression, while qualitative data were analyzed thematically. Lastly, an integrative analysis was undertaken to synthesize and draw inferences from both the quantitative and qualitative findings.

**Findings:**

The satisfaction was recorded as highest in the domain of interpersonal manner (79.54%) and lowest in the domain of accessibility and convenience (32.33%). Medicine availability stood as a strong predictor of patient satisfaction across multiple satisfaction domains which was validated by the qualitative findings. Qualitative findings revealed barriers to provision of healthcare including explanations to the observed levels of satisfaction: limited resources, medicine stock-outs, delays in reimbursement, and ex-post moral hazard among service users.

**Interpretation:**

The study gives insights into both patient-based and provider-based determinants of patient satisfaction. The findings provide recommendations for maintaining an uninterrupted medicine supply chain under the insurance benefit package and necessitate the allocation of enrollees according to the human resource capacity of health institutions which can influence the perceived quality of health services for the long-term sustainability of the insurance program.

## Introduction

The National Health Insurance Program (NHIP) was piloted in 2016 in three districts (*Kailali, Baglung*, and *Ilam*) of Nepal with a political commitment to reduce out-of-pocket expenditure (OOPE) in health. The program covers healthcare services other than free basic health services in order to promote Universal Health Coverage (UHC) [1,2]. The Health Insurance Board (HIB) is the purchaser of health services, and health facilities that fall under the jurisdiction of the three tiers of government (Federal, Provincial and Local levels) are the providers of health insurance services [2]. The program, however, has been facing challenges of low enrollment and high drop-out rates since its establishment and expansion to all 77 districts of the country [3,4]. According to the HIB, 24.7% of the total population was enrolled under the insurance scheme till the year 2022/23 in Nepal [1]. The renewal rate was observed in a decreasing trend with 68% in the year 2021/22, falling from 75% in the preceding year (2020/21) [2]. The renewal rate further decreased to 59% in the year 2022/23 [1]. A structured narrative review conducted to assess organizational and systematic challenges in the implementation of the NHIP reported that the enrollees have been facing compromise in the quality of services received [3]. Additionally, several studies have undertaken efforts to unveil the factors related to enrollment and dropouts of NHIP [4–6]. One of the key causes of dropouts from the NHIP was revealed to be the suboptimal quality of health services received by beneficiaries which directly translated to their satisfaction with health services [6].

It is important to analyze and measure patient satisfaction with national health insurance programs as UHC policies need to be tailored according to the needs and expectations of people. Patient-centered care can only be assured when the gap between the existing health services and expectations of health services to be delivered are assessed and explored before designing insurance programs. Such gaps can compel patients to drop out and find private alternatives resulting in high out-of-pocket expenditure (OOPE) in health, defeating the purpose of the national insurance program [7]. In other words, retention of the insured population depends on the perceived quality of care or level of satisfaction with the health services provided under the benefit package of NHIP. A robust improvement in quality of care is required to sustain enrollment in NHIP which can only be done by identifying the status quo of patient satisfaction along with factors related to service provision influencing their satisfaction levels. In the context of Nepal, there are few studies, at best, that explicitly addresses and measures patient satisfaction under the NHIP [8,9]. Some incorporate specific population group under a limited geographical area [8] while others lack a comprehensive range of factors influencing patient satisfaction, coupled with healthcare provider’s experiences with NHIPs in the NHIP-accredited health facilities [9]. A household survey conducted in *Illam* district of Nepal revealed user satisfaction to be 53.60% [8]. Another recent study conducted to assess satisfaction with the NHIP revealed varying levels of satisfaction under the domains of service utilization (98.90%) and service availability (60.70%) [9]. To the best of our knowledge, there have been no mixed methods explorations including the providers’ perspectives on the perceived quality of health services provisioned under the NHIP.

Public sector hospitals can be hubs for high patient in-flow and subsequent heavy workload causing an environment of distress among providers [10]. A study conducted in Ghana depicted that patient satisfaction can be considerably explained by attitudes of healthcare providers attributed to higher workload and delayed reimbursements under the national health insurance schemes [11]. Another study conducted in a similar regional context suggested that active participation and motivation of health service providers leverage expansion of UHC through social health insurance [12]. On top of that, health service delivery procedures determines the quality of healthcare given to the public [12]. Providers’ viewpoints are crucial for enhancing validity of quantitative satisfaction surveys, ensuring overall accountability to insurance policy-making process and providing feedback for the quality and scope of services covered by health insurance programs. Understanding and gathering perspectives from both patients and further explaining the satisfaction levels by perceived barriers to access health services among providers can effectively help streamline health insurance policies to improve quality of health services and promote higher subscription rates under the NHIP.

Through a mixed methods study triangulating both patient satisfaction surveys and qualitative interviews with healthcare providers, this present study aims to delve into the broader perspectives of satisfaction along with its determinants for health services provided under the insurance benefit package. The study goes on to inform providers’ perspectives on the barriers to provision of healthcare in line with the observed predictors of satisfaction levels in quantitative survey. Finally, the study further integrates both findings to provide holistic and balanced approach that addresses both satisfaction among insured patients and provider-based explanations to patient satisfaction. Such explanations are paramount in understanding the complex contextual and circumstantial factors as well as implementation processes of delivering insurance-related health services in NHIP-listed institutions.

## Materials and methods

### Study design

A cross-sectional, mixed methods study was adopted where 391 insured patients visiting Bhaktapur hospital (a provincial-level NHIP-listed hospital) were surveyed, followed by in-depth interviews with seven healthcare providers for identifying barriers to the provision of quality care influencing patient satisfaction. The study was conducted from 16 October, 2023 to 30 December, 2023. The mixed methods study followed an explanatory sequential design (S1 Fig) as given by Creswell [13], where in quantitative satisfaction survey was followed by qualitative study to explain the results of quantitative study. Among 16 accredited specialists or above allopathic health facilities listed under the national health insurance scheme in Kathmandu Valley, Bhaktapur Hospital was purposively selected since it had a history of providing health services under the national health insurance scheme since 2017 [14]. Along with this, as a referral center, the hospital served patients from different districts all over Nepal. The hospital had a high outpatient flow of insured patients with a total of 90,417 insurance services registered in the year 2021/22 [14]. Cochran’s formula for infinite population was used for the determination of sample size in which the prevalence (P) of patient satisfaction was taken from a study conducted in a tertiary health facility with a similar outpatient department (OPD) setting where the overall patient satisfaction was calculated to be 64% on average [15]. The sample size was calculated using a 5% margin of error and 95% confidence interval. The sample size thus calculated was “354.04 ≅ 355”. A non-response rate of 10% was added to the calculated sample size. Finally, a total of 391 participants were recruited from the OPD patients in Bhaktapur Hospital.

Ethical approval was obtained from the Institutional Review Committee (IRC) of Manmohan Memorial Institute of Health Sciences, affiliated with Tribhuvan University, Institute of Medicine (NEHCO/IRC/080/062; Oct 08, 2023). Administrative approval was obtained from Bhaktapur Hospital for the collection of data. We also obtained a support letter from the HIB, Teku, Kathmandu.

### Participants

For quantitative survey, participants above the age of 18, enrolled in the insurance program for at least six months, and having at least one prior consultation or examination covered by the government insurance scheme were eligible. Respondents who previously discontinued their participation but subsequently re-enrolled were also retained in the analysis as their inclusion would provide critical insights into factors influencing satisfaction. The participants were recruited from the insurance ticket/billing counter, waiting lines, and the pharmacy of the hospital. A purposive sampling technique was used due to the lack of comprehensive administrative lists of insures and frequent referrals from other facilities which were not under the hospital records. Participants were excluded if they were enrolled in the insurance program but visited the facility for services other than those covered by the insurance benefit package.

For the qualitative study, a purposive sample of seven healthcare providers from different outpatient departments was selected based on pre-defined criteria including whether their departments provided health insurance services, their knowledge of the NHIP payment mechanism and their willingness to participate. The healthcare professionals from OPD that had services that were not under insurance benefit package – like Dental OPD and Ophthalmic OPD, were excluded.

Written consent forms were provided to both insured patients and healthcare providers and the participants were ensured that the information collected would remain confidential. A unique serial number was assigned to each questionnaire/interview to ensure anonymity.

### Procedures

The quantitative survey included four major components – sociodemographic characteristics, health and hospital-related factors, insurance-related factors and patient satisfaction. The former three components were measured through a semi-structured questionnaire finalized through multiple iterations by the research team based on extensive literature review and relevancy of the items to the NHIP and the study hospital. The questionnaire was also reviewed by content experts from the insurance board and the hospital. On the other hand, the latter component, ‘patient satisfaction’ was measured through a standard tool, ‘PSQ-18’, developed by RAND Corporation which is available for free in the public domain [16]. PSQ-18 contains a five-point Likert scale (ranging from strongly agree to strongly disagree) consisting of seven dimensions: general satisfaction, technical quality, interpersonal manner, communication, financial aspects, time spent with doctor, and accessibility and convenience [16]. Face-to-face interviews were conducted with six to eight enrollees per day. Paper-based questionnaires were used to collect data covering socio-demographic variables (age, sex, marital status, education status, religion, ethnicity, occupation, income, area of residence, language, family size), hospital and health-related variables (time taken to reach the health facility, self-reported health status, type of illness), and insurance-related variables (willingness to pay (WTP), insurance type, renewal status, perceived benefits of insurance, years of enrollment, knowledge of health insurance program). The data on sex was self-reported by the study participants themselves with options; male, female, others, and prefer not to say. Respondents were asked to provide their individual monthly income instead of household income as many respondents were not able to report total income of the household during pretesting of the study. The definition and set categories for the variables are enclosed in S1 Table.

Pretesting was conducted among 39 participants in a similar accredited hospital providing national health insurance services. Internal homogeneity tests were conducted on the questionnaire tool where Cronbach alpha was calculated to be 0.78 which was above the acceptable limit of 0.7. The reliability score of the tool conducted in similar settings in Nepal ranged from 0.79–0.8 [15,17]. Also, Nepali version of PSQ-18 having reliability score of above 0.8 was collected from previous author [15]. While the tool is yet to be validated in Nepal, it demonstrated validity for use in OPD settings in the Saudian context through assessment of psychometric properties of the tool (where the authors used confirmatory factor analysis for studying structure’s goodness of fit) and an acceptable concurrent validity in similar health care context of Nigeria [18,19].

For qualitative data collection, the approach to inquiry used was based on phenomenological study method by Creswell (2013) [20]. In-depth interviews were conducted to explore the providers’ perspectives on the barriers to healthcare provision affecting patient satisfaction under the NHIP. The interview guidelines containing 18 items were formulated after an extensive literature review [10–12,21–28] incorporating factors like perception on NHIP, interpersonal relationships, communication, payment mechanism of NHIP and overall experiences of the health professionals in service delivery for insured patients which were set as priori codes. The items were later revised to address the major predictors of patient satisfaction identified in the quantitative survey to further explain the essence of the quantitative findings. A full spectrum of providers’ perspectives was explored by including healthcare providers from all OPDs that provided insurance services as shown in (Table 1). Data collection and analysis were done iteratively after each transcript. Data saturation was considered reached in the seventh interview whereby no new codes or themes emerged and responses were repetitive across the OPD departments.

**Table 1:** List of interviews conducted among health service providers.

S.N.	Gender	Position	Type of OPD/ Duty station
1	Female	Gynecology/Obstetric Consultant	Gynecology/ Obstetric OPD
2	Female	Psychiatric Consultant	Psychiatric/Mental OPD
3	Male	Senior Surgeon Consultant	Surgical OPD
4	Male	Pediatric Consultant	Pediatric OPD
5	Male	Physician Consultant	Medical OPD
6	Male	Orthopedic Consultant	Orthopedic OPD
7	Female	Nursing Officer	Administration

All the interview were conducted face-to-face at a private setting of the respective OPDs. The interviews were recorded where consented, and field notes were taken during the interviews. The interview was conducted for an average duration of 30 minutes.

### Statistical analysis

Frequency tables along with percentages were used to describe the study sample. Bivariate analysis was conducted using chi-square test (χ^2^) to assess the association of patient satisfaction across all independent variables. An ordinal logistic regression model was used to assess the factors associated with patient satisfaction. All the statistically significant variables (p < 0.05) from the bivariate chi-square were included in the multivariable model as potential confounders to obtain adjusted odds ratios. While formal tests of model assumptions and model-fits were not performed, the final model was guided by significance in chi-square analysis and prior empirical evidences from the literature review [15,29–31]. SPSS (version 20) was used to analyze the collected data from the paper-based questionnaires. Continuous variables like age and income were converted into categorical variables based on their distribution. Age was categorized into quartile groups and income was dichotomized based on the median value of the sample. Such categorization was applied for rigorous quantitative analysis accounting for potential non-linear relationship to the dependent variable. No mathematical or log transformations were used for continuous variables. Categorical variables like (sex, ethnicity, etc.) were dummy coded for analysis. Similarly, in order to determinate adequacy of knowledge, a three-item knowledge questions were scored and those with scores of two or higher were considered to have ‘adequate knowledge’. The three-item questions were based on the services listed and not listed under NHIP, and the insurance ceiling amount depending on the number of family members enrolled. For details on this, please refer to S2 Table.

In this study, the dependent variable was categorized into three ordinal variables: ‘not satisfied, neutral and satisfied’ by using quartile cut-off points independently for each domain of PSQ-18. This was done considering the data on the midline scores being significantly skewed either to ‘not-satisfied’ or ‘satisfied’ in the absence of ‘neutral’ category which might have otherwise affected the interpretation of the results. Satisfaction was measured separately across the seven domains. Since there was no aggregate satisfaction score, analyses by satisfaction level (satisfied/neutral/not satisfied) were conducted independently for each domain. Domain-specific chi-square test of independence analysis is presented in the S3 Table.

### Qualitative analysis

The audio recordings of the in-depth interviews along with the notes were transcribed and translated into English. A set of priori codes were listed based on the literature reviews of selected qualitative studies [10–12,21–28] which were further revised based on the satisfaction levels and their major co-variates identified in the quantitative survey. The transcripts were analyzed to label additional emerging codes. A codebook (S4 Table) was developed where the codes underwent several iterations and were then categorized into key themes supported by verbatims. Intra-coder reliability was applied to test the agreement scores for reliability. Since the number of the transcripts were limited, all of the transcripts were re-coded after a six-weeks interval where the agreement was found to be higher than 70%. They were analyzed manually through thematic analysis.

### Mixed methods integration

An integrative analysis of both the findings of quantitative and qualitative data was performed which was interpreted using joint analysis table given in S5 Table.

### Role of funding source

There was no funding source for this study.

## Results

### Quantitative findings

#### Characteristics of the participants.

The socio-demographic and insurance-related characteristics of the participants is summarized in (Table 2). The response rate of the survey was 100% (n = 391). Most of the participants belonged to middle age group (40–59 years) and the respondents were predominantly female (67.30%). Most of the participants were married and the majority (91.04%) were followers of the Hindu religion. The majority of the participants had no personal income, largely due to many being homemakers, unemployed, or others including retirees and students. Regarding insurance-related characteristics, almost all (97.95%) reported that insurance premium was affordable and nearly half of the respondents reported that they were willing to pay more than the existing premium amount (NPR 3,500 as of 2024) given that the insurance ceiling was increased along with the availability of better-quality benefit package. Almost two-thirds were enrolled for three or more years. The majority of the respondents (70.84%) reported that medicines were available to them through the health insurance package. It was found that approximately two-thirds of the participants had inadequate knowledge regarding the national health insurance program. A full list of variables, including all categories and subgroup details, is provided in S1 and S2 Tables.

**Table 2:** Characteristics of the study respondents.

	Frequency (%)
**Sociodemographic variables**	
**Age (Mean ± SD**^**a**^ **= 47·09 ± 14·46)**	
18-20	12 (3·07)
21-39	108 (27·62)
40-59	186 (47·57)
60 or above	85 (21·74)
**Sex**	
Male	128 (32·70)
Female	263 (67·30)
**Religion**	
Hindu	356 (91·04)
Buddhist	11 (2·81)
Christian	15 (3·83)
Others	9 (2·29)
**Ethnicity**	
Brahmin/Chhetri	156 (39·89)
Janajati	215 (54·98)
Others	20 (5·1)
**Monthly Income in NPR**^**b**^ **(Mean ± SD**^**a**^ **= 6809·46 ± 35624·82)**	
No income	345 (88·20)
< 35000	21 (5·40)
≥ 35000	25 (6·40)
**Occupation**	
Agriculture	13 (3·30)
Business	25 (6·40)
Homemaker	140 (35·90)
Unemployed	97 (24·90)
Others	116(29.5)
**Hospital and insurance-related variables**	
**Self-Reported Health Status**	
Good	25 (6·39)
Moderate	313 (80·05)
Bad	53 (13·55)
**Type of illness**	
Acute	103 (26·34)
Chronic	288 (73·66)
**NHI premium affordability**	
Affordable	383 (97·95)
Not Affordable	8 (2·05)
**Willingness to pay for higher insurance ceiling and better-quality package**	
Yes	187 (47·95)
No	203 (52·05)
**Years of enrollment (Mean ± SD**^**a**^ **= 3.2 ± 1.75)**	
< 3	142 (36·32)
≥ 3	249 (63·68)
**Availability of medicine**	
Available	277 (70·84)
Unavailable	114 (29·16)
**Overall knowledge of the NHIP**	
Adequate	126 (32·20)
Inadequate	265 (66·67)

^a^Standard Deviation.

^b^Nepalese Rupees.

### Patient satisfaction across seven dimensions of PSQ-18

A wide variation in satisfaction was observed across seven dimensions of PSQ-18. A higher percentage of satisfaction was observed in the domain of interpersonal manner with highest mean satisfaction score, followed by time spent with the doctor, and communication. The satisfaction proportion was found the lowest in the domain of accessibility and convenience (32.33%), followed general satisfaction (36.06%) as illustrated in (**Table 3**).

**Table 3:** Average satisfaction in seven domains of PSQ-18.

Satisfaction Domain	Mean of the satisfaction scores	Standard Deviation	Satisfaction in percentage (Average percentage % of satisfaction in each item)
General Satisfaction	3·26	0·66	36·06
Technical Quality	3·68	0·42	58·82
Interpersonal Manner	3·77	0·49	79·54
Communication	3·68	0·53	69·05
Financial Aspects	3·43	0·77	58·57
Time Spent with Doctor	3·62	0·68	71·61
Accessibility and Convenience	3·14	0·49	32·33

(**Table 4**) illustrates the patient satisfaction by each item of the PSQ-18. Nearly two-thirds of the respondents felt confident that they could get medical care without being financially set back. Almost half of the respondents denied having easy access to medical specialists they required. Additionally, over half of the respondents agreed that they found it hard to receive an appointment for medical care they received.

**Table 4:** Segregation of satisfaction by each item of PSQ-18.

Patient Satisfaction Items	Yes (Strongly agree + Agree) n (%)	Uncertain n (%)	No (Strongly disagree + Disagree) n (%)	Mean	S.D.
1.Doctors are good about explaining the reason for medical tests	350 (89·50)	18 (4·60)	23 (5·90)	2·16	0·515
2.I think my doctor’s office has everything needed to provide complete medical care.	255 (65·20)	25 (6·40)	111 (28·40)	2·63	0·904
3.The medical care I have been receiving is just about perfect.	308 (78·80)	23 (5·90)	60 (15·30)	2·36	0·741
4.Sometimes doctors make me wonder if their diagnosis is correct.	50 (12·80)	24 (6·20)	316 (81·00)	3·68	0·696
5.I feel confident that I can get the medical care I need without being set back financially.	256 (65·50)	50 (12·80)	85 (21·70)	2·56	0·829
6.When I go for medical care, they are careful to check everything when treating and examining me	350 (89·50)	27 (6·90)	14 (3·60)	2·13	0·461
7.I have to pay for more of my medical care than I can afford.	91 (23·30)	47 (12·00)	253 (64·70)	3·41	0·848
8.I have easy access to the medical specialists I need	141 (36·10)	68 (17·40)	182 (46·50)	3·10	0·909
9.Where I get medical care, people have to wait too long for emergency treatment	25 (6·40)	197 (50·40)	169 (43·20)	3·37	0·609
10.Doctors act too businesslike and impersonal toward me	15 (3·80)	58 (14·80)	318 (81·30)	3·77	0·502
11.My doctors treat me in a very friendly and courteous manner	323 (82·60)	46 (11·80)	22 (5·60)	2·23	0·546
12.Those who provide my medical care sometimes hurry too much when they treat me	80 (20·50)	17 (4·30)	294 (75·20)	3·55	0·812
13.Doctors sometimes ignore what I tell them	85 (21·70)	19 (4·90)	287 (73·40)	3·52	0·828
14.I have some doubts about the ability of the doctors who treat me.	33 (8·40)	20 (5·10)	338 (86·40)	3·78	0·584
15.Doctors usually spend plenty of time with me	321 (82·10)	18 (4·60)	52 (13·30)	2·31	0·694
16.I find it hard to get an appointment for medical care I receive	232 (59·30)	25 (6·40)	134 (34·30)	2·74	0·949
17.I am dissatisfied with some things about the medical care I receive	209 (53·50)	21 (5·40)	161 (41·20)	2·87	0·970
18.I am able to get medical care whenever I need it	288 (73·70)	38 (9·70)	65 (16·60)	2·42	0·767

### Association between independent variables with seven domains of patient satisfaction using ordinal logistic regression

The variables found to be associated in chi-square (S3 Table) are illustrated in (Table 5) for each satisfaction domain. Religion, ethnicity, years of enrollment, medicine availability, knowledge of NHIP, marital status, family size, presence of individual income, willingness to pay, time taken to reach the health facility, type of illness, and self-reported health status were among the independent variables found associated with patient satisfaction. The variables were further analyzed using ordinal logistic regression analysis of patient satisfaction where ‘not satisfied’ was taken as the reference category.

**Table 5:** Association of independent variables with seven domains of patient satisfaction.

		Reference category = ‘Not Satisfied’			
		Satisfied		Neutral	
		OR (95% CI)	p-value	OR (95% CI)	p-value
**General Satisfaction** ^**a**^	Type of Illness: Chronic	1·22 [0·74-1·99]	0·435	0·36 [0·15-0·87]	0·023
**Technical Quality**	Marital Status: Married	2·14 [0·88-5·20]	0·095	3·41 [1·20-9·66]	0·021
	Religion: Hindu	3·18 [1·04-9·79]	0·043	1·31 [−·42-4·14]	0·643
	Ethnicity: *Janajati*	1·68 [0·42-6·78]	0·465	2·92 [0·68-12·42]	0·148
	Occupation: Homemakers	2·17 [0·96-4·90]	0·063	1·92 [0·79-4·55]	0·146
	Occupation: Unemployed	3·03 [1·15-7·98]	0·025	2·45[0·88-6·85]	0·086
	Self-Reported Health Status: Good	2·71 [1·13-6·50]	0·025	2·85 [1·08-7·47]	0·034
	WTP: Yes	1·22 [0·60-2·47]	0·578	0·56 [0·26-1·19]	0·132
	Years of Enrollment (in years): ≥ 3	1·23 [0·60-2·53]	0·567	0·92 [0·43-1·97]	0·820
**Interpersonal Manner**	Income: Absent	3·87 [1·38-10·86]	0·010	2·43 [0·74-7·99]	0·144
	Type of Illness: Chronic	2·17 [0·85-5·58]	0·107	1·15 [0·41-3·27]	0·792
**Communication** ^**a**^	Occupation: Homemakers	2·22 [0·74-6·64]	0·153	1·83 [0·58-5·70]	0·300
	Occupation: Unemployed	3·05 [0·82-11·31]	0·096	1·17 [0·29-4·76]	0·826
**Financial Aspect**	Marital Status: Married	2·14 [0·95-4·82]	0·065	1·34 [0·55-3·28]	0·520
	Ethnicity: *Janajati*	1·55 [0·88-2·75]	0·130	2·79 [1·41-5·51]	0·003
	Family size: ≤ 5	0·98 [0·54-1·76]	0·932	1·84 [0·87-3·89]	0·110
	Availability of medicines: Available	4·06 [2·30-7·17]	<0·001	2·83 [1·44-5·53]	0·002
**Time spent with doctor**	Income: Absent	3·19 [1·51-6·77]	0·002	2·95 [1·03-8·45]	0·044
	Years of enrollment: ≥ 3	1·83 [0·99-3·36]	0·052	1·04 [0·49-2·22]	0·916
**Accessibility and Convenience**	Income: Absent	0·89 [0·35-2·32]	0·820	0·39 [0·16-0·91]	0·028
	Type of Illness: Chronic	1·92 [1·08-3·42]	0·026	2·36 [1·34-4·15]	0·003
	Medicine Availability: Available	2·62 [1·46-4·70]	0·001	1·67 [0·98-2·85]	0·061
	Knowledge of National Health Insurance Program: Adequate	2·19 [1·22-3·92]	0·009	1·29 [0·72-2·33]	0·381

OR= Adjusted Odds Ratio. WTP = Willingness to Pay. Only variables significant in the crude (unadjusted) analysis are displayed for conciseness; all significant variables from bivariate analysis were included in the multivariate model. For detailed table, please refer to S6 Table. ‘Not satisfied’ was used as the reference category for the dependent variable. Each independent variable has its own reference category specified and odds ratio is presented as the likelihood of being in a higher satisfaction category compared with ‘Not satisfied’, relative to that of independent variable’s reference category. The reference group for categorical variables: acute for type of illness, ≥ 60 for age, unmarried for marital status, non-Hindu for religion, others for ethnicity, others for occupation, < 3 for years of enrollment, bad for self-reported health status, no for willingness to pay, present for income, unavailable for medicine availability, inadequate for knowledge of national health insurance program, and >5 for family size. The confounders adjusted for each variable include: age, marital status, religion, ethnicity, mother language, occupation, time taken to reach health facility, self-reported health status, WTP, and years of enrollment for technical quality, income, and type of illness for interpersonal manner, marital status, religion, ethnicity, family size, occupation, years of enrollment, and availability of medicines for financial aspect, income, and years of enrollment for time spent with doctor and ethnicity, mother language, income, type of illness, medicine availability, and knowledge of health insurance program for accessibility and convenience. All of the confounders adjusted had bivariate p < 0.05.

^a^Crude Odds ratio displayed (due to a single significant variable under the domain whereby no confounding was adjusted for the satisfaction domain)

In terms of religion, the odds of Hindu participants reporting to be satisfied was 3.18 times [AOR: 3.18, CI = 1.04–9.79] that of their non-Hindu counterparts under the domain of technical quality. The study also showed that the odds of satisfaction among those unemployed was 3.03 times the odds [AOR: 3.03, CI = 1.15–7.98] of those who belonged to certain occupational groups. Compared to those who reported their health status to be ‘bad’, the odds of satisfaction among respondents who reported their health status to be ‘good’ was 2.71 times higher. [AOR: 2.71, CI = 1.13–6.50]

Under the domain of interpersonal manner, the odds of satisfaction among the participants who did not have an individual income was 3.87 times [AOR: 3.87, CI = 1.38–10.86] that of the participants having an individual income. The study found no association of the independent variables with communication except for occupation as presented by (**Table 5**). In terms of medicine availability, the odds of satisfaction among those who reported that medicines were available was 4.06 times [AOR: 4.06, CI = 2.30–7.17] times that of the respondents who reported that medicines were unavailable under the domain of financial aspect.

In the domain of time spent with doctor, the odds of satisfaction among respondents who enrolled for 3 years or more was 1.83 times [AOR: 1.83, CI = 0.99–3.36] that of respondents who enrolled for less than 3 years. Additionally, compared to those having an individual income, those who did not have an individual income had higher odds [AOR: 3.19, CI = 1.51–6.77] of being satisfied.

Regarding the type of illness, the odds of the respondents who reported having a chronic condition were 1.92 times [AOR: 1.92, CI = 1.08–3.42] that of the respondents who reported to have an acute condition under the domain of accessibility and convenience. The odds of respondents who reported that medicines were available were 2.62 times [AOR: 2.62, CI = 1.46–4.70] that of the respondents who reported that medicines were unavailable. The respondents who had adequate knowledge regarding the national health insurance program had higher odds [AOR: 2.19, CI = 1.22–3.92] of being satisfied than respondents who had inadequate knowledge of the national health insurance program.

The quantitative findings warranted a further in-depth qualitative finding under the insurance-related characteristics like medicine availability, knowledge of the NHIP and types of illnesses among insured patients which remained statistically significant after ordinal logistic regression. Additionally, patient satisfaction domains including time spent with doctor (71.61%), technical quality (58.82%) or resource availability, accessibility and convenience (32.33%) also underwent qualitative exploration in relation to prior literature review [11,12,26,29] and services directly provided by healthcare providers in OPD.

### Qualitative findings

The detailed themes and codes as per thematic analysis are presented in S4 Table. Six major themes are briefly outlined in the following sub-sections:

### Issues with medicine quality and availability

Concerns over the quality of the medicines provided under NHIP’s standard list of medicine were reported along with frequent medicine stock-outs. This prevented the insured patients from experiencing optimum satisfaction that they otherwise would have felt. The stock-outs of medicine further aggravates and complicates the single-day validation of prescription of the insured patients. Healthcare providers mentioned that medicine availability was one of the main factors that resulted in greater patient satisfaction. In line with this, medicine availability revealed to be a strong predictor of patient satisfaction (p < 0.001) in the quantitative survey and was found associated with multiple domains of patient satisfaction like financial aspect and accessibility and convenience.

*“There is no consistency in the quality of the medicines. Sometimes, we receive good quality medicines while other times, we receive poor quality medicines. The medicines also sometimes get exchanged with an alternative medicine [brand] of the same name.”*

-A healthcare consultant, male [M06]

*“When we run out of the insurance medicine stock, patients get disappointed after having to wait for hours in line without getting the medicines they were prescribed with.”*

-A hospital administrator, female [F07]

### Moral hazard

The service providers reported patient-side moral hazard involving the unnecessary demand of prescriptions and misuse of consultations of tests and procedures. Lack of understanding of insurance risk pooling mechanism was considered at the root of patient-side moral hazard. In order to tackle this issue, providers suggested use of co-payment where patients are obliged to pay about 20% or more of their medical bills.

*“About 70-80% of the patients who visit here [the hospital] are genuine cases in need of medical assistance and care. The remaining 25-30% of patients misuse the services and visit for the sake of receiving unnecessary consultations, tests, and medications. Patients visit just for the sake of wanting more medicines added to their prescription paper.”*

-A healthcare consultant, male [M04]

### Challenges with the reimbursement system

Reimbursement of insurance services proved critical for the hospital. However, this was reportedly affected by reimbursement delays and discrepancies, server glitches and defined timeframe for claim to be realized.

*“The [hospital] reimbursement is not done promptly and the exact amount reimbursed is sometimes not received.”*

-A healthcare consultant, male [M03]

*“In some circumstances, the server goes down, and we sometimes cannot claim reimbursement amounts on time. If the documents uploaded [for reimbursement] have any mistakes, then the hospital has to bear the loss of medicines dispensed for free. There should be one door system for uploading the documents which is currently unavailable… we have to claim insurance amount every single day. However, if we cannot claim for up to one week, such reimbursement can no longer be applicable and it gets nullified.”*

-A hospital administrative officer, female [F07]

### De-incentivization/demotivation among the service providers

The quantitative findings show that the majority of the patients reported that the doctors could provide enough time for consultation (82.10%). However, most healthcare providers reported that they attended to an overwhelming number of patients each day at the OPD forcing them to limit consultation time. They reported that a tedious referral system further increased their work burden which was already entrenched with human resource deficit. The NHIP did not have any provisions to provide capitation or fee for services for health service providers treating insured patients.

*“We see up to 120-130 patients every day. We have to offer consultations to the patients in a rush. On top of that, we attend both the emergency OT and the OPD which further adds to our work burden... The hospital patient inflow has doubled since the [insurance] program started but the human resources are present in the same number which overburdens the entire hospital system… The referral system could also improve so that they will not be need lengthy paperwork. It could be digitalized.”*

-A healthcare consultant, male [M03]

### Lack of coordinated roll-out of health insurance services

Designation of the hospital as the service contact point without considering the hospital resource capacity was deemed as a miscalculation while rolling out the health insurance program. The providers reported lack of streamlined monitoring visits as well as coordination between health insurance board and accredited health facilities in regards to operating health insurance services and refresher trainings for health professionals.

*“The outpatient flow especially after the health insurance program has increased a lot. This has not been taken into consideration with respect to the capacity of the hospital and the health workers currently available. “*

-A healthcare consultant, female [F01]

*The insurance board can play a key role in coordinating, and take action when referrals are frequently asked even when the notices for one time referral requirements are issued about what issues are leading to this.”*

-A hospital administrative officer, female [F07]

### Time spent with doctor

Long-term visitors reportedly had improved relationships with doctors. However, short consultation time to accommodate high patient inflow reportedly jeopardized patient satisfaction for most patients may prefer longer consultation periods.

*“We are required to give about 5 minutes in each consultation with our patients. However, practically that is not always possible… the health service that we cater right now is more quantity-based than quality based and we need to attend to every patient regardless of the time they are getting for the consultation.”*

-A healthcare consultant, female [F01]

### Mixed methods findings

The joint display table (S5 Table) presents the integrated findings where insurance-related characteristics and satisfaction domains were explained using the follow-up qualitative study and inferences were drawn from the integrated analysis. These inferences are further discussed in the discussion section along with relevant theory under different literatures.

### Insurance-related characteristics

The quantitative findings indicated that the medicines were not available at all times with almost one-third reporting medicine unavailability. This was also evident in the qualitative results explaining the reason being frequent stock out of insurance-listed medications in the hospitals forcing the patients to buy medicines from private pharmacies, compromising their satisfaction. Similarly, almost half of the respondents reported willingness to pay higher for the insurance premium. This can be complemented by the findings in qualitative interviews where the need to curb moral hazard through the use of co-payment was reported.

### Satisfaction domain

Almost a quarter of the respondents were satisfied in the domain of ‘Time spent with doctor’ as per the quantitative results. Qualitatively, the providers reported not being able to provide enough time for consultation due to high patient inflow. On contrast, it was also reported that they had good interpersonal relationship with older patients who have been visiting them for a longer period of time which was observed to influence patient satisfaction.

Also, insured patients reported less satisfaction under the domain of accessibility and convenience. Qualitatively, the unavailability of specialist services in OPD and difficulty in referral processes explains the lower satisfaction. Approximately 20% of the respondents reported that the providers sometimes rush their treatment processes as reported in the quantitative findings. As per the service providers, mixed reactions were observed from the patients when the providers were more straightforward and assertive during the consultations during rush hours. This is likely to have influenced the satisfaction experienced by the patients.

### Knowledge of the NHIP

As reported in the quantitative findings, respondents with adequate knowledge of the NHIP had higher odds of being satisfied in comparison to respondents with inadequate knowledge. The qualitative findings provided insight into the differences in satisfaction attributed to the knowledge of the NHIP. It was reported that the knowledge of insurance risk pooling functions along with the insurance provisions determines the conscious and rational use of insurance services. It was also reported that the lack of knowledge and negligence by the insured patients lead to missed deadlines for reimbursement of the hospital services.

## Discussion

Our analysis of the cross-sectional dataset showed that NHIP enrollees had a wide range of satisfaction levels under different satisfaction domains of the study. Our findings show the least satisfaction under the domain of accessibility and convenience (32.33%) where attributing items like patients’ access to specialist care, and ease in getting an appointment for medical care had high disagreement rates. This can be explained by the high patient inflow and lack of specialist services in the OPD of the hospital under study. Some evidence suggests that health insurance does not necessarily improve access to health care for all [32,33]. Access itself has a complex web of determinants like effectiveness of the service provided, social class and economic class of people, distance, acceptance of provider, and financial barriers [34]. In our study, enrollees were more satisfied under the domains of interpersonal manner and time spent with doctor which remains consistent with previous findings from similar settings and contexts [15,17]. Despite most services being covered free of cost under the insurance program, the study found patient satisfaction under the domain of financial aspect to be relatively lower in comparison to a previous study [15]. This can be attributed to the frequent unavailability of medicines as reported in qualitative findings, compelling patients to buy medicines at high prices from pharmacies.

Our study revealed various challenges in health service delivery under the NHIP: medicine unavailability, demand-side moral hazard, delays in reimbursement, human resource deficit, tedious referral process, and lack of information on reimbursement time periods among NHIP enrolled patients. While supply-side moral hazard might be rarely existent in public health facilities as physicians are not currently incentivized under the NHIP, practices of taking unnecessary tests and medicines were widespread as reported by healthcare providers. Such behavior can impact and present as an obstacle to the operation and conscious use of collective fund under the government health insurance program. The main reason insurance is considered valuable is because it transfers consumption from those who are healthy to those who are sick and in need of care [35,36]. These factors can be responsible for dissatisfaction among the healthcare providers which might affect the quality of health services offered ultimately deciding satisfaction among their patients [36].

The majority of the patients found the NHI premium affordable, and almost half of them reported willing to pay more than the then existing premium amount under the given conditions of better-quality health services and higher ceiling offered in the program. Evidence show that patients have lower WTP for medication and higher WTP for non-communicable diseases (NCDs) [37]. In line with this, the qualitative results highlighted the need of co-payment mechanisms to curb unnecessary use of health insurance services. Willingness to Pay, medicine availability, income status and type of illness were found significantly associated with patient satisfaction in our study. This highlights the need to consider medicine access, income and illness status when designing premiums and determining the extent of services offered under the benefit package of NHIP.

### Implications for public health and research

In our study, it was revealed that the respondents who had adequate knowledge regarding the national health insurance program had higher odds of being satisfied compared to neutral and not satisfied than respondents who had inadequate knowledge of the national health insurance program. Similarly, in a study regarding enrollee satisfaction with basic medical insurance in China, one of the key predictors of patient satisfaction was the awareness of insurance policies including premiums and compensation among the patients [38]. A systematic review and meta-analysis on factors affecting voluntary uptake of community-based health insurance in low and middle-income countries suggests that the knowledge and understanding of insurance can act as an enabler for enrolment [30]. Meanwhile, a poor understanding of insurance policies can act as a barrier to the renewal of insurance [30]. The qualitative findings supported the fact that inadequate knowledge of risk pooling functions can lead to misuse and unnecessary demands of medicine prescriptions. Public awareness programs need to be upscaled country-wide, especially regarding the premium amount, risk pooling, changes in policy provisions, and the services enlisted under the benefit package.

Medicine availability was seen as an important predictor of patient satisfaction in our study where participants who reported that medicines were available had higher odds of being satisfied under the domains of financial aspect and accessibility and convenience. This result is consistent with a previous study conducted in various health facilities of the *Kaski* district of Nepal in a similar OPD setting where it was found that insured patients who reported that medicines were available were more likely to be satisfied than those who did not receive medicine at health facilities [39]. Another study conducted at the household level in *Illam* district of Nepal also revealed that the respondents who received prescribed medicines were up to four times more likely to be satisfied than those who did not receive prescribed medicines regularly [8]. This was further validated in the qualitative findings with providers reporting that patients were dissatisfied during periods of medicine stock outs. This consistency in findings across multiple studies highlights the need for maintaining a proper medicine supply chain to prevent any stock-outs of medicines. This can help improve the patient’s perception of quality healthcare services and encourage them to continue their enrolment in the program.

Years of enrollment stood as an important influencing factor for patient satisfaction where the odds of respondents enrolled for three years or more to be satisfied was two times that of respondents who those enrolled for less than three years under the domain of time spent with doctor. Individuals enrolled will continue to subscribe to the health insurance program if they perceive the health care services to be of the highest possible quality [40]. Studies conducted in Nigeria and Pakistan have shown that patients who have been enrolled for a longer period have a better perception of the insurance scheme [12, 41]. The results of the qualitative study also suggest that frequent consultative visits improve the interpersonal relationship with healthcare providers resulting in better satisfaction among insured patients, particularly those of older age groups. Thus, it is necessary to prevent dropouts and introduce quality improvement tools for enhancing the quality of insurance services to retain the enrollees and appeal the program to wider population groups.

### Strengths and limitations of the study

The mixed methods approach complementing the patient satisfaction survey with provider’s perspectives of barriers to provision of health services under the NHIP provided useful insights into the overall landscape of the implementation status and challenges of the health insurance scheme. The study included a large sample size that improved its capacity to run statistical tests and assess the association of satisfaction with a wide range of variables. The study examined both patient-based and provider-side factors that influence patient satisfaction which was rarely dealt with before by previous studies of such nature [8,9]. Despite this, the study also had some limitations. The study undertook purposive sampling method to select insured patients due to unavailability of sampling frame of insured patients which limited generalizability of the findings. The study was conducted before some significant policy changes took place in referral mechanisms and medicine prescription periods. So, there could be differences in the level of patient satisfaction after insurance policy changes were implemented. The study was conducted in a closed institutional setting with respondents which could lead to social desirability bias with respondents over/underreporting their satisfaction or dissatisfaction with the health services. The study has limited generalizability in primary level health facilities considering differences in technical capacity and human resources. The study could not explore in-depth perspectives from insured patients with subjective experiences and expectations underlying the satisfaction level which can be further incorporated in future studies. Another limitation in the study was that model assumptions and best model fit tests for the ordinal logistic regression were not formally tested. Further tests could include model fit tests to enhance model robustness. Additionally, since our study computed odds ratio as a metric for measuring strength of association, this could limit interpretability among general readers. Future studies could compute predicted probabilities or marginal effects to facilitate dissemination of results to non-technical stakeholders. The small proportion of respondents with prior drop-out experiences might result in overestimation of satisfaction since the study primarily included current enrollees. This might lead to selection bias as satisfaction levels among insurance users who permanently discontinued enrollment may differ from those reported in this study.

Further studies comparing patient satisfaction among insured population across different tiers of health facilities from super-specialty hospitals to primary health facilities can help uncover wider determinants of patient satisfaction. Studies can incorporate health facility-related factors like seating arrangements, environmental sanitation, drinking water availability, and behavior of hospital support staff which might influence patient satisfaction. Also, continued efforts should be made to conduct more research on the topic to facilitate secondary reviews to further inform the national insurance policies and programs that can contribute to promoting health equity and improve access to healthcare.

## Conclusion

There were wide discrepancies observed across the domains of patient satisfaction in this study. Patient satisfaction was recorded highest in the domain of interpersonal manner and lowest in the domain of accessibility and convenience despite the insurance program being introduced to bridge the gap in access. This can be attributed to the lack of health specialists in the hospital outpatient department, medicine unavailability, tedious referral process, and limited infrastructure in the hospital under study. The study underscores the need to maintain a proper supply chain to prevent medicine stockouts and to expand patient education initiatives to improve renewal rates and retention of insured patients. Significant improvements in policy including provider-based concerns like the doctor-patient ratio, especially in the context of insured patients, and technical issues with reimbursements are crucial considering the changing scenario of health service delivery at hospitals. Strengthened coordination among the health ministry, policymakers, and healthcare professionals is needed to devise holistic plans to address limited infrastructure and human resource capacities to improve the overall quality of care in health facilities. Thus, the findings serve as critical input for quality improvement efforts for policymakers and the hospital under study.

## Supporting information

S1 Fig Procedural Diagram: An explanatory, sequential mixed methods. This diagram shows procedures and products of each phase horizontally, adapted from Ivankova, Creswell and Stick (2006).
(TIF)

S1 Table Variables table with categorization. The table shows how each variable is operationally defined as per relevant references.
(DOCX)

S2 Table Descriptive Variables. This table includes all independent variables that could not be included in the main text.
(DOCX)

S3 Table Domain-specific chi-square test of significance analysis. Bivariate Analysis Table (Chi-square test of independence) for each of the seven domains under PSQ-18 tool.
(DOCX)

S4 Table Codebook. Codebook for detailed qualitative findings.
(DOCX)

S5 Table Joint Table. A joint table analysis of quantitative and follow-up qualitative findings regarding insurance-related characteristics and patient satisfaction.
(DOCX)

S6 Table Ordinal Logistic Regression Analysis. The full table showing analysis in detailed form.
(DOCX)

S1 Appendix Questionnaire and Interview Guidelines. Includes all the items asked during the satisfaction survey and the key-informant interview guidelines.
(DOCX)

S1 Data Full quantitative dataset of satisfaction survey. (SAV)
