# Supplementary material for: Patient satisfaction among national health insurance enrollees in an accredited hospital of Kathmandu Valley: A cross-sectional, mixed methods study
Source: PLoS One. 2026 Mar 20;21(3):e0345353. doi: 10.1371/journal.pone.0345353 (PMC13004337; doi:10.1371/journal.pone.0345353)
Supplement: S1 Table — The table shows how each variable is operationally defined as per relevant references. (DOCX) [file pone.0345353.s002.docx]

**S1 Table. Variables table with categorization.** The table shows how each variable is operationally defined as per relevant references.

| **SN** | **Variables** | **Definition/Categories** | **Category** |
| --- | --- | --- | --- |
| 1. | Patient satisfaction | It is the degree/level of satisfaction as measured by the drawing satisfaction scores from the subscales as defined in the PSQ-18 tool. | Dependent Variable |
| 2. | Willingness to Pay | It is calculated as the maximum (non-zero) amount that individuals are willing to pay for insurance scheme [1]. | Independent Variable |
| 3. | Chronic Illness | Participants who get an illness symptom lasting for more than 6 months from the data collection period [1]. | Independent Variable |
| 4. | Knowledge of NHIP | Three-item questions were asked to assess the knowledge of patients of the insurance program. The three items had similar weights. Each right response received a score of 1 while each wrong response received a score of 0. The total score would fall between the score 0 to 3. The knowledge of the participants with score 2 or higher was deemed adequate, otherwise inadequate [1]. | Independent Variable |
| 5. | Premium affordability | It refers to the ability to pay or the affordability to pay without bearing a heavy financial burden [2]. | Independent Variable |
| 6. | Insurance type | It defines the type of insurance plan which may be categorized as subsidized or non-subsidized. | Independent Variable |
| 7. | Sex (assigned at birth) | Male, Female, Prefer not to say, Others (please specify) | Independent Variable |
| 8. | Religion | Hindu, Buddhist, Muslim, Kirat, Christian, Others (Please specify) as listed in tool of Nepal Demographic Health Survey (NDHS) [3]. | Independent Variable |
| 9. | Caste/Ethnicity | Brahmin, Chhetri, Dalit, Janajati, Muslim, Others (Please specify) modified as listed in tool of Nepal Demographic Health Survey (NDHS) [3]. | Independent Variable |
| 10. | Native language | Nepali, Maithili, Bhojpuri, Tharu, Newari, Tamang, Others (Please specify) | Independent Variable |
| 11. | Self-Reported Health Status | Very good, Good, Moderate, Bad, Very Bad as listed in the tool of Nepal Demographic Health Survey (NDHS) [3]. |  |
| 12. | Area of Residence | Urban area (Metropolitan or Sub-Metropolitan or Municipality), Rural area (Rural Municipality) as listed in the tool of Nepal Demographic Health Survey (NDHS) [3]. |  |

**References:**

[1] Girmaw F, Adane E, Kassaw AT, Ashagrie G, Baye T. Willingness to Pay for Social Health Insurance Among Health Care Professionals in North Wollo Zone, Amhara Region, Ethiopia: Mixed Method Study. Clin Outcomes Res CEOR [Internet]. 2023 [cited 2023 Sep 12];15:593. Available from: https:/pmc/articles/PMC10387270/

[2] Russell S. Ability to pay for health care: concepts and evidence. Health Policy Plan [Internet]. 1996 [cited 2023 Sep 12];11(3):219–37. Available from: <https://pubmed.ncbi.nlm.nih.gov/10160370/>

[3] Ministry of Health and Population, Nepal; New ERA; and ICF. 2022. Nepal Demographic and Health Survey 2022: Key Indicators Report. Kathmandu, Nepal: Ministray of Health and Population, Nepal. Available from: dhsprogram.com/pubs/pdf/PR142/PR142.pdf
