# Supplementary material for: Patient satisfaction among national health insurance enrollees in an accredited hospital of Kathmandu Valley: A cross-sectional, mixed methods study
Source: PLoS One. 2026 Mar 20;21(3):e0345353. doi: 10.1371/journal.pone.0345353 (PMC13004337; doi:10.1371/journal.pone.0345353)
Supplement: S2 Table — This table includes all independent variables that could not be included in the main text. (DOCX) [file pone.0345353.s003.docx]

**S2 Table. Descriptive Variables.** This table includes all independent variables that could not be included in the main text.

| **Individual Characteristics** | **Frequency (%)** |
| --- | --- |
| **Educational Status** |  |
| Cannot read/write | 85 (21.70) |
| Primary Education (1 to 8) | 108 (27.60) |
| Secondary Education (9 to 12) | 135 (34.50) |
| Tertiary Education (Bachelor and above) | 63 (16.10) |
| **Type of Insurance** |  |
| Subsidized | 27 (6.91) |
| Not Subsidized | 364 (93.09) |
| **Marital Status** |  |
| Unmarried | 39 (10·00) |
| Married | 343 (87·70) |
| Widow/Widower | 9 (2·30) |
| **Religion** |  |
| Hindu | 356 (91·04) |
| Buddhist | 11 (2·81) |
| Kirat | 4 (1·02) |
| Christian | 15 (3·83) |
| Others | 5 (1·27) |
| **Ethnicity** |  |
| Brahmin | 98 (25·06) |
| Chhetri | 58 (14·83) |
| Dalit | 9 (2·30) |
| Janajati | 215 (54·98) |
| Madhesi | 9 (2·30) |
| Others | 2 (0·50) |
| **Occupation** |  |
| Agriculture | 13 (3·30) |
| Business | 25 (6·40) |
| Government Service | 10 (2·60) |
| Private Service | 57 (14·60) |
| Labor | 1 (0·30) |
| Student | 20 (5·10) |
| Homemaker | 140 (35·90) |
| Retired | 27 (6·90) |
| Unemployed | 97 (24·90) |
| **Native Language** |  |
| Nepali | 183 (46·80) |
| Maithili | 4 (1·02) |
| Bhojpuri | 2 (0·51) |
| Newari | 173 (44·25) |
| Tamang | 21 (5·37) |
| Others (including Rai, Magar, and Limbu) | 8 (2.05) |
| **Family Size** |  |
| ≤5 | 272 (69·60) |
| >5 | 119 (30·40) |
| **Time to reach the hospital (in minutes)** |  |
| <30 | 218 (55·75) |
| 30-60 | 137 (35·04) |
| >60 | 36 (9·21) |
| **Amount willing to pay (in NPR)** |  |
| 4000-5000 | 118 (63·44) |
| >5000 | 68 (36·56) |
| **Annual renewal of insurance** |  |
| Yes | 354 (95·40) |
| No | 15 (4·10) |
| **Knowledge about provisions under NHIP** |  |
| Yes | 320 (81·80) |
| No | 71 (18·20) |
| **Knowledge about services under the benefit package** |  |
| Yes | 261 (66·80) |
| No | 130 (33·20) |
| **Knowledge about services not under the benefit package** |  |
| Yes | 37 (9·50) |
| No | 354 (90·54) |

| **Benefits of the insurance program** | **Frequency (%)** |
| --- | --- |
| **Do you think insurance program provides you with benefits?** | |
| Yes | 383 (98.00) |
| No | 8 (2.00) |
| **Benefits of the insurance program** |  |
| Prevents unexpected healthcare expenditure | 272 (43.03) |
| Benefits those who cannot afford treatment cost | 348 (55.06) |
| Timely treatment | 8 (1.26) |
| Increase in Quality of health services | - - 1. (0.63) |

| **Renewal Status of Insurance** | **Frequency (%)** |
| --- | --- |
| **Have you renewed your insurance status every year since enrollment?** | |
| Yes | 354 (95.40) |
| No | 15 (4.10) |
| **Reasons for non-renewal of insurance** |  |
| Did not consider it necessary | 3 (20.00) |
| Missed the renewal dates/ Renewal got overlooked | 2 (13.33) |
| Could not contact insurance agent for renewal | 2 (13.33) |
| Did not know the process of renewal | 1 (6.67) |
| Long distance from the hospital | 1 (6.67) |
| Unavailability of medicines | 6 (40.00) |

| **Knowledge about Insurance Program** | **Frequency (%)** |
| --- | --- |
| **Do you know about the provisions under the national health insurance program?** | |
| Yes | 320 (81.80) |
| No | 71 (18.20) |
| **Insurance provisions** |  |
| Annual Subscription of insurance premium of Rs.3,500 in a family of 5 members | 317 (99.70) |
| Insurance program runs on a voluntary mechanism | 15 (4.70) |
| Ceiling of benefit package is up to 1,00,000 and adding Rs. 700 with every member added to the insurance package | 135 (42.50) |
| Insured get services from primary health facilities and private and community government hospital through referral | 5 (1.60) |

| **Knowledge about Insurance Program** | **Frequency (%)** |
| --- | --- |
| **Do you know of service under the benefit package?** |  |
| Yes | 261 (66.80) |
| No | 130 (33.20) |
| **Services under the benefit package** |  |
| OPD | 124 (95.40) |
| Emergency Services | 36 (27.70) |
| IPD | 26 (20.00) |
| Diagnostic Services | 79 (60.80) |
| Listed Medicines | 50 (38.50) |
| Surgery | 20 (15.40) |
| Glasses, hearing aid, white stick, and crutches at any rate | 2 (1.50) |

| **Knowledge about Insurance Program** | **Frequency (%)** |
| --- | --- |
| **Do you know the services that are not under the benefit package?** |  |
| Yes | 37 (9.50) |
| No | 354 (90.54) |
| **Services not under the benefit package** |  |
| Prescription/vision glasses, hearing aid, white stick, and crutches at more than the determined rate | 17 (47.20) |
| Plastic and cosmetic surgery | 8 (22.20) |
| Surgery for burns, cleft lip and palate, and serious forms of disability | 2 (5.60) |
| Dental treatment except for primary management of dental extraction, dental abscess, and dental trauma | 20 (55.60) |
