# Supplementary material for: Patient satisfaction among national health insurance enrollees in an accredited hospital of Kathmandu Valley: A cross-sectional, mixed methods study
Source: PLoS One. 2026 Mar 20;21(3):e0345353. doi: 10.1371/journal.pone.0345353 (PMC13004337; doi:10.1371/journal.pone.0345353)
Supplement: S3 Table — Bivariate Analysis Table (Chi-square test of independence) for each of the seven domains under PSQ-18 tool. (DOCX) [file pone.0345353.s004.docx]

**S3 Table. Domain-specific chi-square test of significance analysis.** Bivariate Analysis Table (Chi-square test of independence) for each of the seven domains under PSQ-18 tool.

### **Chi-square test of independence**

**Association between general satisfaction and socio-demographic characteristics**

| **Characteristics** | **General Satisfaction** | | | **Chi-square value** | **p-value** |
| --- | --- | --- | --- | --- | --- |
|  | **Satisfied (%)** | **Neutral (%)** | **Not Satisfied (%)** |  |  |
| **Age** |  |  |  |  |  |
| <40 | 39 (32·50) | 12 (10·00) | 69 (57·50) | 7·26 | 0·122 |
| 40-57 | 63 (35·80) | 7 (3·98) | 106 (60·23) |  |  |
| ≥ 58 | 39 (41·05) | 3 (3·16) | 53 (55·79) |  |  |
| **Sex** |  |  |  |  |  |
| Male | 42(32·81) | 7 (5·47) | 79 (61·72) | 0·94 | 0·624 |
| Female | 99(37·64) | 15 (5·70) | 149 (56·65) |  |  |
| **Marital Status** |  |  |  |  |  |
| Unmarried | 12 (25·00) | 6 (12·50) | 30 (62·50) | 6·615 | 0·037 (F) |
| Married | 129 (37·60) | 16 (4·66) | 198 (57·73) |  |  |
| **Religion** |  |  |  |  |  |
| Hindu | 130 (36·51) | 17 (4·78) | 209 (58·71) | 5·458 | 0·065 (F) |
| Non-Hindu | 11 (31·42) | 5 (14·29) | 19 (54·29) |  |  |
| **Ethnicity** |  |  |  |  |  |
| Janajati | 75 (34·88) | 8 (3·72) | 132 (61·40) | 4·045 | 0·132 |
| Others | 66 (37·50) | 14 (4·95) | 96 (54·55) |  |  |

**F = Fisher’s Exact Test.**

**Association between general satisfaction and family size, language and area of residence**

| **Characteristics** | **General Satisfaction** | | | **Chi-square value** | **p-value** |
| --- | --- | --- | --- | --- | --- |
|  | **Satisfied (%)** | **Neutral (%)** | **Not Satisfied (%)** |  |  |
| **Native Language** |  |  |  |  |  |
| Nepali | 71 (38·79) | 14 (7·65) | 98 (53·55) | 4·55 | 0·103 |
| Non-Nepali | 70 (33·65) | 8 (3·85) | 130 (62·50) |  |  |
| **Area of Residence** |  |  |  |  |  |
| Rural/Urban Municipality | 124 (36·47) | 19 (5·59) | 197 (57·94) | 1·89 | 0·910 |
| Sub-metropolitan/Metropolitan | 17 (33·33) | 3 (5·88) | 31 (60·78) |  |  |
| **Family Size** |  |  |  |  |  |
| ≤5 | 99 (36·39) | 17 (6·25) | 156 (57·35) | 0·786 | 0·675 |
| >5 | 42 (35·29) | 5 (4·20) | 72 (60·50) |  |  |

**Association between general satisfaction and education and economy related characteristics**

| **Characteristics** | **General Satisfaction** | | | **Chi-square value** | **p-value** |
| --- | --- | --- | --- | --- | --- |
|  | **Satisfied (%)** | **Neutral (%)** | **Not Satisfied (%)** |  |  |
| **Educational Status** |  |  |  |  |  |
| Illiterate | 35 (41·18) | 2 (2·35) | 48 (56·47) | 2·92 | 0·232 (F) |
| Literate | 106 (34·64) | 20 (6·54) | 180 (58·82) |  |  |
| **Income** |  |  |  |  |  |
| Present | 14 (30·43) | 2 (4·35) | 30 (65·22) | 1·03 | 0·596 (F) |
| Absent | 127 (36·81) | 20 (5·80) | 198 (57·39) |  |  |
| **Occupation** |  |  |  |  |  |
| Homemaker | 61 (43·57) | 5 (3·57) | 74 (52·86) | 3·10 | 0·780 |
| Unemployed | 32 (32·99) | 4 (4·12) | 61 (62·89) |  |  |
| Others | 48 (31·37) | 13 (8·50) | 92 (60·13) |  |  |

**F = Fisher’s Exact Test.**

**Association between general satisfaction with hospital and health related characteristics**

| **Characteristics** | **General Satisfaction** | | | **Chi-square value** | **p-value** |
| --- | --- | --- | --- | --- | --- |
|  | **Satisfied (%)** | **Neutral (%)** | **Not Satisfied (%)** |  |  |
| **Time taken to reach health facility (in minutes)** | | | |  |  |
| <30 | 85 (38·99) | 15 (6·88) | 118 (54·13) | 4·272 | 0·370 |
| 30-60 | 44 (32·11) | 5 (3·65) | 88 (64·23) |  |  |
| >60 | 12 (33·33) | 2 (5·56) | 22 (61·11) |  |  |
| **Self-reported Health Status** | |  |  |  |  |
| Good | 122 (36·09) | 20 (5·92) | 196 (57·99) | 0·42 | 0·811 (F) |
| Bad | 19 (35·84) | 2 (3·77) | 32 (60·38) |  |  |
| **Type of Illness*** |  |  |  |  |  |
| Acute | 32 (31·10) | 11 (10·68) | 60 (58·25) | 7·313 | 0·026 |
| Chronic | 109 (37·84) | 11 (3·82) | 168 (58·33) |  |  |

**F = Fisher’s Exact Test. *significantly associated (< 0.05).**

**Association between general satisfaction and insurance related characteristics**

| **Characteristics** |  | | **General Satisfaction** | | **Chi-square value** | **p-value** |
| --- | --- | --- | --- | --- | --- | --- |
|  | **Satisfied (%)** | **Neutral (%)** | | **Not Satisfied (%)** |  |  |
| **Health Insurance Premium Affordability** | | | | |  |  |
| Affordable | 140 (36·55) | 21 (5·48) | | 222 (57·96) | 2·33 | 0·311 |
| Not Affordable | 1 (12·50) | 1 (12·50) | | 6 (75·00) |  |  |
| **Benefit from Insurance** | |  | |  |  |  |
| Yes | 140 (36·55) | 22 (5·74) | | 221 (57·70) | 2·91 | 0·233 (F) |
| No | 1 (12·50) | 0 (0·00) | | 7 (87·50) |  |  |
| **Willingness to Pay for greater insurance ceiling and better-quality service package** | | | | | | |
| Yes | 68 (36·36) | 6 (3·21) | | 113 (60·43) | 3·93 | 0·140 (F) |
| No | 73 (35·96) | 16 (7·88) | | 114 (56·16) |  |  |
| **WTP (in NRP)** |  |  | |  |  |  |
| 4000-4500 | 48 (40·67) | 5 (4·24) | | 65 (55·08) | 2·36 | 0·124 |
| ≥ 5000 | 20 (29·41) | 1 (1·47) | | 47 (69·12) |  |  |
| **Type of Insurance** |  |  | |  |  |  |
| Subsidized | 13 (48·14) | 2 (7·41) | | 12 (44·44) | 2·29 | 0·317 (F) |
| Not Subsidized | 128 (35·16) | 20 (5·49) | | 216 (59·34) |  |  |
| **Years of enrollment (in years)** | |  | |  |  |  |
| <3 | 41 (28·87) | 10 (7·04) | | 91 (64·08) | 5·26 | 0·072 |
| ≥ 3 | 100 (40·16) | 12 (4·82) | | 137 (55·02) |  |  |
| **Renewed insurance every year** | |  | |  |  |  |
| Yes | 133 (37·57) | 19 (5·37) | | 202 (57·06) | 2·08 | 0·352 (F) |
| No | 4 (26·66) | 2 (13·33) | | 9 (60·00) |  |  |
| **Availability of medicines** | |  | |  |  |  |
| Available | 108 (39·98) | 14 (5·05) | | 155 (55·96) | 3·72 | 0·156 |
| Unavailable | 33 (28·94) | 8 (7·02) | | 73 (64·04) |  |  |
| **Knowledge of NHIP** |  |  | |  |  |  |
| Adequate | 47 (37·30) | 8 (6·35) | | 71 (56·35) | 0·37 | 0·829 |
| Inadequate | 94 (35·47) | 14 (5·28) | | 157 (59·25) |  |  |

**F = Fisher’s Exact Test. NPR = Nepalese Rupees. NHIP = National Health Insurance Program.**

**Association between technical quality and social demographic characteristics**

| **Characteristics** | **Technical Quality** | | | **Chi-square value** | **p-value** |
| --- | --- | --- | --- | --- | --- |
|  | **Satisfied (%)** | **Neutral (%)** | **Not Satisfied (%)** |  |  |
| **Age*** |  |  |  |  |  |
| <40 | 72 (60·00) | 28 (23·33) | 20 (16·67) | 9·71 | 0·046 |
| 40-57 | 100 (56·82) | 62 (35·23) | 14 (7·95) |  |  |
| ≥ 58 | 58 (61·05) | 23 (24·21) | 14 (14·74) |  |  |
| **Sex** |  |  |  |  |  |
| Male | `66 (51·56) | 43 (33·59) | 19 (14·84) | 4·18 | 0·124 |
| Female | 164 (62·36) | 70 (26·62) | 29 (11·03) |  |  |
| **Marital Status*** |  |  |  |  |  |
| Unmarried | 25 (52·08) | 9 (18·75) | 14 (29·17) | 15·01 | 0·001 |
| Married | 205 (59·77) | 104 (30·32) | 34 (9·91) |  |  |
| **Religion*** |  |  |  |  |  |
| Hindu | 217 (60·96) | 99 (27·81) | 40 (11·24) | 8·22 | 0·016 |
| Non-Hindu | 13 (37·14) | 14 (40·00) | 8 (22·86) |  |  |
| **Ethnicity*** |  |  |  |  |  |
| Janajati | 140 (65·12) | 56 (26·05) | 19 (8·84) | 9·16 | 0·010 |
| Others | 90 (51·14) | 57 (32·39) | 29 (16·48) |  |  |

***significantly associated (< 0.05)**

**Association between technical quality and family size, language and area of residence**

| **Characteristics** | **Technical Quality** | | | **Chi-square value** | **p-value** |
| --- | --- | --- | --- | --- | --- |
|  | **Satisfied (%)** | **Neutral (%)** | **Not Satisfied (%)** |  |  |
| **Native Language*** |  |  |  |  |  |
| Nepali | 91 (49·73) | 63 (34·43) | 29 (15·85) | 12·05 | 0·002 |
| Non-Nepali | 139 (66·83) | 50 (24·04) | 19 (9·13) |  |  |
| **Area of Residence** |  |  |  |  |  |
| Rural/Urban Municipality | 206 (60·59) | 95 (27·94) | 39 (11·47) | 3·59 | 0·166 |
| Sub-metropolitan/Metropolitan | 24 (47·06) | 18 (35·29) | 9 (17·65) |  |  |
| **Family Size** |  |  |  |  |  |
| ≤ 5 | 159 (58·46) | 73 (26·84) | 40 (14·71) | 5·63 | 0·060 |
| >5 | 71 (59·66) | 40 (33·61) | 8 (6·72) |  |  |

***significantly associated (< 0.05)**

**Association between technical quality and education and economic characteristics**

| **Characteristics** | **Technical Quality** | | | **Chi-square value** | **p-value** |
| --- | --- | --- | --- | --- | --- |
|  | **Satisfied (%)** | **Neutral (%)** | **Not Satisfied (%)** |  |  |
| **Educational Status** |  |  |  |  |  |
| Illiterate | 57 (67·06) | 22 (25·88) | 6 (7·06) | 4·01 | 0·135 |
| Literate | 173 (56·54) | 91 (29·74) | 42 (13·73) |  |  |
| **Income (Individual)** |  |  |  |  |  |
| Present | 26 (56·52) | 11 (23·91) | 9 (19·57) | 2·75 | 0·253 |
| Absent | 204 (59·13) | 102 (29·57) | 39 (11·30) |  |  |
| **Occupation*** |  |  |  |  |  |
| Homemaker | 87 (62·14) | 41 (29·29) | 12 (8·57) | 10·94 | 0·027 |
| Unemployed | 62 (63·92) | 28 (28·87) | 7 (7·22) |  |  |
| Others | 80 (52·29) | 44 (28·76) | 29 (18·95) |  |  |

***significantly associated (< 0.05)**

**Association between technical quality and hospital and health related characteristics**

| **Characteristics** | **Technical Quality** | | | **Chi-square value** | **p-value** |
| --- | --- | --- | --- | --- | --- |
|  | **Satisfied (%)** | **Neutral (%)** | **Not Satisfied (%)** |  |  |
| **Time taken to reach health facility (in minutes)*** | | | |  |  |
| <30 | 144 (66·06) | 52 (23·85) | 22 (10·09) | 15·863 | 0·003 |
| 30-60 | 63 (45·99) | 50 (36·50) | 24 (17·52) |  |  |
| >60 | 23 (63·89) | 11 (30·56) | 2 (5·56) |  |  |
| **Self-reported Health Status*** | |  |  |  |  |
| Good | 204 (60·36) | 98 (28·99) | 36 (10·65) | 6·37 | 0·041 |
| Bad | 26 (49·06) | 15 (28·30) | 12 (22·64) |  |  |
| **Type of Illness** |  |  |  |  |  |
| Acute | 68 (66·02) | 23 (22·33) | 12 (11·65) | 3·36 | 0·186 |
| Chronic | 162 (56·25) | 90 (31·25) | 36 (12·50) |  |  |

***significantly associated (< 0.05)**

**Association between technical quality and insurance related characteristics**

| **Characteristics** | **Technical Quality** | | | **Chi-square value** | **p-value** |
| --- | --- | --- | --- | --- | --- |
|  | **Satisfied (%)** | **Neutral (%)** | **Not Satisfied (%)** |  |  |
| **Health Insurance Premium Affordability** | | | |  |  |
| Affordable | 226 (59·01) | 110 (28·72) | 47 (12·27) | 0·32 | 0·853 (F) |
| Not Affordable | 4 (50·00) | 3 (37·50) | 1 (12·50) |  |  |
| **Benefit from Insurance** | | |  |  |  |
| Yes | 227 (59·27) | 111 (28·98) | 45 (11·75) | 4·91 | 0·086 (F) |
| No | 3 (37·50) | 2 (25·00) | 3 (37·50) |  |  |
| **Willingness to pay for a higher ceiling and better-quality service package*** | | | | | |
| Yes | 95 (50·80) | 68 (36·36) | 24 (12·83) | 11·46 | 0·003 |
| No | 135 (66·50) | 44 (21·67) | 24 (11·82) |  |  |
| **WTP (in NPR)** |  |  |  |  |  |
| 4000-4500 | 60 (50·85) | 45 (38·14) | 13 (11·02) | 1·12 | 0·572 |
| ≥5000 | 34 (50·00) | 23 (33·82) | 11 (16·18) |  |  |
| **Type of Insurance** |  |  |  |  |  |
| Subsidized | 17 (62·96) | 9 (33·33) | 1 (3·70) | 2·02 | 0·365 (F) |
| Not Subsidized | 213 (58·52) | 104 (28·57) | 47 (12·91) |  |  |
| **Years of enrollment (in years)*** | |  |  |  |  |
| <3 | 72 (50·70) | 47 (33·10) | 23 (16·20) | 6·65 | 0·036 |
| ≥3 | 158 (63·45) | 66 (26·51) | 25 (10·04) |  |  |
| **Renewed insurance every year** | | | |  |  |
| Yes | 210 (59·32) | 105 (29·66) | 39 (11·02) | 0·66 | 0·719 (F) |
| No | 10 (66·67) | 3 (20·00) | 2 (13·33) |  |  |
| **Availability of medicines** | |  |  |  |  |
| Available | 164 (59·21) | 78 (28·16) | 35 (12·64) | 0·30 | 0·859 |
| Unavailable | 66 (57·89) | 35 (30·70) | 13 (11·40) |  |  |
| **Knowledge of NHIP** |  |  |  |  |  |
| Adequate | 73 (57·94) | 36 (28·57) | 17 (13·49) | 0·26 | 0·880 |
| Inadequate | 157 (59·25) | 77 (29·06) | 31 (22·70) |  |  |

**F = Fisher’s Exact Test. WTP = Willingness to Pay. NPR = Nepalese Rupees. NHIP = National Health Insurance Program. *significantly associated (< 0.05).**

**Association between interpersonal manner and socio-demographic characteristics**

| **Characteristics** | **Interpersonal Manner** | | | **Chi-square value** | **p-value** |
| --- | --- | --- | --- | --- | --- |
|  | **Satisfied (%)** | **Neutral (%)** | **Not Satisfied (%)** |  |  |
| **Age** |  |  |  |  |  |
| <40 | 87 (72·50) | 25 (20·83) | 8 (6·67) | 7·48 | 0·112 |
| 40-57 | 144 (81·82) | 26 (14·77) | 6 (3·41) |  |  |
| ≥ 58 | 80 (84·21) | 9 (9·47) | 6 (6·32) |  |  |
| **Sex** |  |  |  |  |  |
| Male | 103 (80·47) | 19 (14·84) | 6 (4·69) | 0·12 | 0·942 |
| Female | 208 (79·09) | 41 (15·59) | 14 (5·32) |  |  |
| **Marital Status** |  |  |  |  |  |
| Unmarried | 31 (64·58) | 15 (31·25) | 2 (4·17) | 10·65 | 0·005 (F) |
| Married | 280 (81·63) | 45 (13·12) | 18 (5·25) |  |  |
| **Religion** |  |  |  |  |  |
| Hindu | 284 (79·78) | 53 (14·89) | 19 (5·34) | 0·95 | 0·621 (F) |
| Non-Hindu | 27 (77·14) | 7 (20·00) | 1 (2·86) |  |  |
| **Ethnicity** |  |  |  |  |  |
| Janajati | 176 (81·86) | 30 (13·95) | 9 (4·19) | 1·73 | 0·421 |
| Others | 135 (76·70) | 30 (17·05) | 11 (6·25) |  |  |

**F = Fisher’s Exact Test. *significantly associated (< 0.05).**

**Association between interpersonal manner and family size, language and area of residence**

| **Characteristics** | **Interpersonal Manner** | | | **Chi-square value** | **p-value** |
| --- | --- | --- | --- | --- | --- |
|  | **Satisfied (%)** | **Neutral (%)** | **Not Satisfied (%)** |  |  |
| **Native Language** |  |  |  |  |  |
| Nepali | 139 (75·96) | 32 (17·49) | 12 (6·56) | 2·98 | 0·225 |
| Non-Nepali | 172 (82·69) | 28 (13·46) | 8 (3·85) |  |  |
| **Area of Residence** |  |  |  |  |  |
| Rural/Urban Municipality | 206 (60·59) | 95 (27·94) | 39 (11·47) | 4·73 | 0·094 |
| Sub-metropolitan/Metropolitan | 24 (47·06) | 18 (35·29) | 9 (17·65) |  |  |
| **Family Size** |  |  |  |  |  |
| ≤ 5 | 217 (79·78) | 41 (15·07) | 14 (5·15) | 0·051 | 0·975 |
| >5 | 94 (78·99) | 19 (15·97) | 6 (5·04) |  |  |

***significantly associated (< 0.05)**

**Association between interpersonal manner and education and economic characteristics**

| **Characteristics** | **Interpersonal Manner** | | | **Chi-square value** | **p-value** |
| --- | --- | --- | --- | --- | --- |
|  | **Satisfied (%)** | **Neutral (%)** | **Not Satisfied (%)** |  |  |
| **Educational Status** |  |  |  |  |  |
| Illiterate | 57 (67·06) | 22 (25·88) | 6 (7·06) | 1·16 | 0·560 |
| Literate | 173 (56·54) | 91 (29·74) | 42 (13·73) |  |  |
| **Income*** |  |  |  |  |  |
| Present | 26 (56·52) | 11 (23·91) | 9 (19·57) | 7·98 | 0·019 |
| Absent | 204 (59·13) | 102 (29·57) | 39 (11·30) |  |  |
| **Occupation** |  |  |  |  |  |
| Homemaker | 87 (62·14) | 41 (29·29) | 12 (8·57) | 0·036 | 0·982 |
| Unemployed | 62 (63·92) | 28 (28·87) | 7 (7·22) |  |  |
| Others | 80 (52·29) | 44 (28·76) | 29 (18·95) |  |  |

***significantly associated (< 0.05)**

**Association between interpersonal manner and hospital and health related characteristics**

| **Characteristics** | **Interpersonal Manner** | | | **Chi-square value** | **p-value** |
| --- | --- | --- | --- | --- | --- |
|  | **Satisfied (%)** | **Neutral (%)** | **Not Satisfied (%)** |  |  |
| **Time taken to reach health facility (in minutes)** | | | |  |  |
| <30 | 172 (78·90) | 36 (16·51) | 10 (4·59) | 1·014 | 0·908 |
| 30-60 | 109 (79·56) | 20 (14·60) | 8 (5·84) |  |  |
| >60 | 30 (83·33) | 4 (11·11) | 2 (5·56) |  |  |
| **Self-reported Health Status** | |  |  |  |  |
| Good | 268 (79·29) | 52 (15·38) | 18 (5·33) | 0·24 | 0·888 (F) |
| Bad | 43 (81·13) | 8 (15·09) | 2 (3·77) |  |  |
| **Type of Illness*** |  |  |  |  |  |
| Acute | 73 (70·87) | 22 (21·36) | 8 (7·77) | 6·539 | 0·038 |
| Chronic | 238 (82·64) | 38 (13·19) | 12 (4·17) |  |  |

**F = Fisher’s Exact Test. *significantly associated (< 0.05).**

**Association between interpersonal manner and insurance related characteristics**

| **Characteristics** | **Interpersonal Manner** | | | **Chi-square value** | **p-value** |
| --- | --- | --- | --- | --- | --- |
|  | **Satisfied (%)** | **Neutral (%)** | **Not Satisfied (%)** |  |  |
| **Health Insurance Premium Affordability** | | | |  |  |
| Affordable | 305 (79·63) | 58 (15·14) | 20 (5·22) | 0·935 | 0·627 (F) |
| Not Affordable | 6 (75·00) | 2 (25·00) | 0 (0·00) |  |  |
| **Benefit from Insurance** | | |  |  |  |
| Yes | 306 (79·90) | 57 (14·88) | 20 (5·22) | 3·32 | 0·189 (F) |
| No | 5 (62·50) | 3 (37·50) | 0 (0·00) |  |  |
| **Willingness to pay for a higher ceiling and better-quality service package** | | | | | |
| Yes | 143 (76·47) | 31 (16·58) | 13 (6·95) | 3·07 | 0·215 |
| No | 167 (82·27) | 29 (14·29) | 7 (3·45) |  |  |
| **WTP (in NPR)** |  |  |  |  |  |
| 4000-4500 | 92 (77·97) | 21 (17·80) | 5 (4·24) | 3·86 | 0·145 (F) |
| ≥5000 | 50 (73·53) | 10 (14·71) | 8 (11·76) |  |  |
| **Type of Insurance** |  |  |  |  |  |
| Subsidized | 22 (81·48) | 3 (11·11) | 2 (7·41) | 0·65 | 0·722 (F) |
| Not Subsidized | 289 (79·40) | 57 (15·66) | 18 (4·95) |  |  |
| **Years of enrollment (in years)** | |  |  |  |  |
| <3 | 108 (76·06) | 26 (18·31) | 8 (5·63) | 1·74 | 0·420 |
| ≥3 | 203 (81·53) | 34 (13·65) | 12 (4·82) |  |  |
| **Renewed insurance every year** | | | |  |  |
| Yes | 282 (79·66) | 54 (15·25) | 18 (5·08) | 1·81 | 0·405 (F) |
| No | 14 (93·33) | 1 (6·67) | 0 (0·00) |  |  |
| **Availability of medicines** | |  |  |  |  |
| Available | 224 (80·87) | 38 (13·72) | 15 (5·42) | 2·02 | 0·365 |
| Unavailable | 87 (76·32) | 22 (19·30) | 5 (4·39) |  |  |
| **Knowledge of NHIP** |  |  |  |  |  |
| Adequate | 98 (77·78) | 20 (15·87) | 8 (6·35) | 0·660 | 0·719 |
| Inadequate | 213 (80·38) | 40 (15·09) | 12 (4·53) |  |  |

**F = Fisher’s Exact Test. WTP = Willingness to Pay. NPR = Nepalese Rupees. NHIP = National Health Insurance Program.**

**Association between Communication and socio-demographic variables**

| **Characteristics** | **Communication** | | | **Chi-square value** | **p-value** |
| --- | --- | --- | --- | --- | --- |
|  | **Satisfied (%)** | **Neutral (%)** | **Not Satisfied (%)** |  |  |
| **Age** |  |  |  |  |  |
| <40 | 81 (67·50) | 36 (30·00) | 3 (2·50) | 7·64 | 0·106 |
| 40-57 | 119 (67·61) | 49 (27·84) | 8 (4·55) |  |  |
| ≥ 58 | 70 (73·68) | 17 (17·89( | 8 (8·42) |  |  |
| **Sex** |  |  |  |  |  |
| Male | 91 (71·09) | 28 (21·88) | 9 (7·03) | 3·26 | 0·196 |
| Female | 179 (68·06) | 74 (28·14) | 10 (3·80) |  |  |
| **Marital Status** |  |  |  |  |  |
| Unmarried | 30 (62·50) | 15 (31·25) | 3 (6·25) | 1·12 | 0·572 (F) |
| Married | 240 (69·97) | 87 (25·36) | 16 (4·66) |  |  |
| **Religion** |  |  |  |  |  |
| Hindu | 252 (70·79) | 88 (24·72) | 16 (4·49) | 5·67 | 0·059 (F) |
| Non-Hindu | 18 (51·43) | 14 (40·00) | 3 (8·57) |  |  |
| **Ethnicity** |  |  |  |  |  |
| Janajati | 153 (71·16) | 54 (25·12) | 8 (3·72) | 1·75 | 0·416 |
| Others | 117 (66·47) | 48 (27·27) | 11 (6·25) |  |  |

**F = Fisher’s Exact Test.**

**Association between communication and family size, language and area of residence**

| **Characteristics** | **Communication** | | | **Chi-square value** | **p-value** |
| --- | --- | --- | --- | --- | --- |
|  | **Satisfied (%)** | **Neutral (%)** | **Not Satisfied (%)** |  |  |
| **Native Language** |  |  |  |  |  |
| Nepali | 119 (65·03) | 53 (28·96) | 11 (6·01) | 2·84 | 0·242 |
| Non-Nepali | 151 (72·60) | 49 (23·56) | 8 (3·85) |  |  |
| **Area of Residence** |  |  |  |  |  |
| Rural/Urban Municipality | 238 (70·00) | 86 (25·29) | 16 (4·71) | 1·09 | 0·579 (F) |
| Sub-metropolitan/Metropolitan | 32 (62·75) | 16 (31·37) | 3 (5·88) |  |  |
| **Family Size** |  |  |  |  |  |
| ≤5 | 184 (67·65) | 72 (26·47) | 16 (5·88) | 2·23 | 0·328 |
| >5 | 86 (72·27) | 30 (25·21) | 3 (2·52) |  |  |

**F = Fisher’s Exact Test.**

**Association between communication and education and economic characteristics**

| **Characteristics** | **Communication** | | | **Chi-square value** | **p-value** |
| --- | --- | --- | --- | --- | --- |
|  | **Satisfied (%)** | **Neutral (%)** | **Not Satisfied (%)** |  |  |
| **Educational Status** |  |  |  |  |  |
| Illiterate | 62 (72·94) | 20 (23·53) | 3 (3·53) | 0·91 | 0·636 (F) |
| Literate | 208 (67·97( | 82 (26·80) | 16 (5·23) |  |  |
| **Income** |  |  |  |  |  |
| Present | 29 (63·04) | 12 (26·09) | 5 (10·87) | 4·15 | 0·126 (F) |
| Absent | 241 (69·86) | 90 (26·09) | 14 (4·06) |  |  |
| **Occupation*** |  |  |  |  |  |
| Homemaker | 96 (68·57) | 39 (27·86) | 5 (3·57) | 11·69 | 0·020 |
| Unemployed | 79 (81·44) | 15 (15·46) | 3 (3·09) |  |  |
| Others | 95 (62·09) | 47 (30·72) | 11 (7·19) |  |  |

**F = Fisher’s Exact Test. *significantly associated (< 0.05).**

**Association between communication and hospital and health related characteristics**

| **Characteristics** | **Communication** | | | **Chi-square value** | **p-value** |
| --- | --- | --- | --- | --- | --- |
|  | **Satisfied (%)** | **Neutral (%)** | **Not Satisfied (%)** |  |  |
| **Time taken to reach health facility (in minutes)** | | | |  |  |
| <30 | 155 (71·10) | 53 (24·31) | 10 (4·59) | 3·73 | 0·443 |
| 30-60 | 87 (63·50) | 42 (30·66) | 8 (5·84) |  |  |
| >60 | 28 (77·77) | 7 (19·44) | 1 (2·78) |  |  |
| **Self-reported Health Status** | |  |  |  |  |
| Good | 238 (69·82) | 87 (25·74) | 15 (4·44) | 1·24 | 0·538 |
| Bad | 34 (64·15) | 15 (28·30) | 4 (7·55) |  |  |
| **Type of Illness** |  |  |  |  |  |
| Acute | 70 (67·96) | 29 (28·16) | 4 (3·88) | 0·53 | 0·768 |
| Chronic | 200 (69·44) | 73 (23·25) | 15 (5·21) |  |  |

**Association between communication and insurance related characteristics**

| **Characteristics** | **Communication** | | | **Chi-square value** | **p-value** |
| --- | --- | --- | --- | --- | --- |
|  | **Satisfied (%)** | **Neutral (%)** | **Not Satisfied (%)** |  |  |
| **Health Insurance Premium Affordability** | | | |  |  |
| Affordable | 263 (68·67) | 102 (26·63) | 18 (4·70) | 3·51 | 0·173 (F) |
| Not Affordable | 7 (87·50) | 0 (0·00) | 1 (12·50) |  |  |
| **Benefit from Insurance** | |  |  |  |  |
| Yes | 265 (69·19) | 99 (25·85) | 19 (4·96) | 0·86 | 0·652 (F) |
| No | 5 (62·50) | 3 (37·50) | 0 (0·00) |  |  |
| **Willingness to Pay for higher insurance ceiling and better-quality service package** | | | | | |
| Yes | 126 (67·37) | 52 (27·81) | 9 (4·81) | 0·51 | 0·775 |
| No | 143 (70·44) | 50 (24·63) | 10 (4·93) |  |  |
| **WTP (in NPR)** |  |  |  |  |  |
| 4000-4500 | 84 (71·19) | 29 (24·58) | 5 (4·24) | 1·76 | 0·415 (F) |
| ≥5000 | 42 (61·76) | 22 (32·35) | 4 (5·88) |  |  |
| **Type of Insurance** |  |  |  |  |  |
| Subsidized | 22 (81·48) | 5 (18·52) | 0 (0·00) | 2·69 | 0·260 (F) |
| Not Subsidized | 248 (68·13) | 97 (26·65) | 19 (5·22) |  |  |
| **Years of enrollment (in years)** | |  |  |  |  |
| <3 | 91 (64·08) | 45 (31·69) | 6 (4·23) | 3·67 | 0·160 |
| ≥3 | 179 (71·88) | 57 (22·89) | 13 (5·22) |  |  |
| **Renewed insurance every year** | | | |  |  |
| Yes | 249 (70·33) | 86 (24·29) | 19 (5·37) | 1·31 | 0·520 (F) |
| No | 10 (66·66) | 5 (33·33) | 0 (0·00) |  |  |
| **Availability of medicines** | |  |  |  |  |
| Available | 190 (68·59) | 73 (26·35) | 14 (5·05) | 0·13 | 0·937 |
| Unavailable | 80 (70·18) | 29 (25·44) | 5 (4·39) |  |  |
| **Knowledge of NHIP** |  |  |  |  |  |
| Adequate | 83 (65·87) | 33 (26·19) | 10 (7·94) | 3·89 | 0·143 |
| Inadequate | 187 (70·56) | 69 (26·04) | 9 (3·40) |  |  |

**F = Fisher’s Exact Test. WTP = Willingness to Pay. NPR = Nepalese Rupees. NHIP = National Health Insurance Program.**

**Association between financial aspects and socio-demographic characteristics**

| **Characteristics** | **Financial Aspects** | | | **Chi-square value** | **p-value** |
| --- | --- | --- | --- | --- | --- |
|  | **Satisfied (%)** | **Neutral (%)** | **Not Satisfied (%)** |  |  |
| **Age** |  |  |  |  |  |
| <40 | 72 (60·00) | 23 (19·17) | 25 (20·83) | 3·66 | 0·454 |
| 40-57 | 96 (54·55) | 45 (25·57) | 35 (19·89) |  |  |
| ≥ 58 | 61 (64·21) | 20 (21·05) | 14 (14·74) |  |  |
| **Sex** |  |  |  |  |  |
| Male | 73 (57·03) | 32 (25·00) | 23 (17·97) | 0·69 | 0·707 |
| Female | 156 (58·31) | 56 (21·29) | 51 (19·39) |  |  |
| **Marital Status*** |  |  |  |  |  |
| Unmarried | 20 (41·67) | 14 (29·17) | 14 (29·17) | 6·78 | 0·034 |
| Married | 209 (60·93) | 74 (21·57) | 60 (17·49) |  |  |
| **Religion*** |  |  |  |  |  |
| Hindu | 215 (60·39) | 73 (20·51) | 68 (19·10) | 9·40 | 0·009 |
| Non-Hindu | 14 (40·00) | 15 (42·86) | 6 (17·14) |  |  |
| **Ethnicity*** |  |  |  |  |  |
| Janajati | 126 (58·60) | 57 (26·51) | 32 (14·88) | 7·53 | 0·023 |
| Others | 103 (58·52) | 31 (17·61) | 42 (23·86) |  |  |

***significantly associated (< 0.05).**

**Association between financial aspects and family size, language and area of residence**

| **Characteristics** | **Financial Aspects** | | | **Chi-square value** | **p-value** |
| --- | --- | --- | --- | --- | --- |
|  | **Satisfied (%)** | **Neutral (%)** | **Not Satisfied (%)** |  |  |
| **Native Language** |  |  |  |  |  |
| Nepali | 105 (57·38) | 35 (19·13) | 43 (23·50) | 5·53 | 0·060 |
| Non-Nepali | 124 (59·62) | 53 (25·48) | 31 (14·90) |  |  |
| **Area of Residence** |  |  |  |  |  |
| Rural/Urban Municipality | 205 (60·29) | 72 (21·18) | 63 (18·53) | 3·59 | 0·166 |
| Sub-metropolitan/Metropolitan | 24 (47·06) | 16 (31·37) | 11 (21·57) |  |  |
| **Family Size*** |  |  |  |  |  |
| ≤5 | 151 (55·51) | 71 (26·10) | 50 (18·38) | 6·69 | 0·035 |
| >5 | 78 (65·55) | 17 (14·29) | 24 (20·17) |  |  |

***significantly associated (< 0.05).**

**Association between financial aspects and education and economy related characteristics**

| **Characteristics** | **Financial Aspects** | | | **Chi-square value** | **p-value** |
| --- | --- | --- | --- | --- | --- |
|  | **Satisfied (%)** | **Neutral (%)** | **Not Satisfied (%)** |  |  |
| **Educational Status** |  |  |  |  |  |
| Illiterate | 46 (54·12) | 18 (21·18) | 21 (24·71) | 2·37 | 0·306 |
| Literate | 183 (59·80) | 70 (22·88) | 53 (17·32) |  |  |
| **Income** |  |  |  |  |  |
| Present | 21 (45·65) | 15 (32·61) | 10 (21·74) | 4·07 | 0·131 |
| Absent | 208 (60·29) | 73 (21·16) | 64 (18·55) |  |  |
| **Occupation*** |  |  |  |  |  |
| Homemaker | 89 (63·57) | 26 (18·57) | 25 (17·86) | 9·87 | 0·043 |
| Unemployed | 64 (65·98) | 16 (16·49) | 17 (17·53) |  |  |
| Others | 76 (49·67) | 45 (29·41) | 32 (20·92) |  |  |

***significantly associated (< 0.05).**

**Association between financial aspects and hospital and health related characteristics**

| **Characteristics** | **Financial Aspects** | | | **Chi-square value** | **p-value** |
| --- | --- | --- | --- | --- | --- |
|  | **Satisfied (%)** | **Neutral (%)** | **Not Satisfied (%)** |  |  |
| **Time taken to reach health facility (in minutes)** | | | |  |  |
| <30 | 131 (60·09) | 54 (24·77) | 33 (15·14) | 8·23 | 0·083 |
| 30-60 | 73 (53·28) | 30 (21·90) | 34 (24·82) |  |  |
| >60 | 25 (69·44) | 4 (11·11) | 7 (19·44) |  |  |
| **Self-reported Health Status** | | | |  |  |
| Good | 205 (60·65) | 74 (21·89) | 59 (17·46) | 5·11 | 0·078 |
| Bad | 24 (45·28) | 14 (26·42) | 15 (28·30) |  |  |
| **Type of Illness** |  |  |  |  |  |
| Acute | 58 (56·31) | 29 (28·16) | 16 (15·53) | 2·95 | 0·228 |
| Chronic | 171 (59·38) | 59 (20·49) | 58 (20·14) |  |  |

**Association between financial aspects and insurance related characteristics**

| **Characteristics** | **Financial Aspects** | | | **Chi-square value** | **p-value** |
| --- | --- | --- | --- | --- | --- |
|  | **Satisfied (%)** |  | **Not Satisfied (%)** |  |  |
| **Health Insurance Premium Affordability** | | | |  |  |
| Affordable | 277 (59·27) | 84 (21·93) | 72 (18·80) | 4·47 | 0·107 (F) |
| Not Affordable | 2 (25·00) | 4 (50·00) | 2 (25·00) |  |  |
| **Benefit from Insurance** | | | |  |  |
| Yes | 227 (59·27) | 86 (22·45) | 70 (18·28) | 5·76 | 0·056 (F) |
| No | 2 (25·00) | 2 (25·00) | 4 (50·00) |  |  |
| **Willingness to Pay for higher insurance ceiling and better-quality service package** | | | | | |
| Yes | 111 (59·36) | 42 (22·46) | 34 (18·18) | 0·08 | 0·960 |
| No | 118 (58·13) | 46 (22·26) | 39 (19·21) |  |  |
| **WTP (in NPR)** |  |  |  |  |  |
| 4000-4500 | 73 (61·86) | 27 (22·88) | 18 (15·25) | 1·39 | 0·497 |
| ≥5000 | 38 (55·88) | 15 (22·06) | 15 (22·06) |  |  |
| **Type of Insurance** |  |  |  |  |  |
| Subsidized | 17 (62·96) | 4 (14·81) | 6 (22·22) | 1·03 | 0·599 |
| Not Subsidized | 212 (58·24) | 84 (23·08) | 68 (18·68) |  |  |
| **Years of enrollment (in years)** | |  |  |  |  |
| <3 | 67 (47·18) | 44 (30·99) | 31 (21·83) | 13·05 | 0·001 |
| ≥3 | 162 (65·06) | 44 (17·67) | 43 (17·27) |  |  |
| **Renewed insurance every year** | |  |  |  |  |
| Yes | 209 (59·04) | 78 (22·03) | 67 (18·93) | 0·04 | 0·981 (F) |
| No | 9 (60·00) | 3 (20·00) | 3 (20·00) |  |  |
| **Availability of medicines*** | |  |  |  |  |
| Available | 180 (64·98) | 62 (22·38) | 35 (12·64) | 26·54 | <0·001 |
| Unavailable | 49 (42·98) | 26 (22·81) | 39 (34·21) |  |  |
| **Knowledge of NHIP** |  |  |  |  |  |
| Adequate | 70 (55·56) | 31 (24·06) | 25 (19·84) | 0·73 | 0·693 |
| Inadequate | 159(60·00) | 57 (21·51) | 49 (18·49) |  |  |

**F = Fisher’s Exact Test. WTP = Willingness to Pay. NPR = Nepalese Rupees. NHIP = National Health Insurance Program. *significantly associated (< 0.05).**

**Association between time spent with doctor and socio-demographic variables**

| **Characteristics** | **Time Spent with Doctor** | | | **Chi-**  **square value** | **p-value** |
| --- | --- | --- | --- | --- | --- |
|  | **Satisfied (%)** | **Neutral (%)** | **Not Satisfied (%)** |  |  |
| **Age** |  |  |  |  |  |
| <40 | 82 (68·33) | 21 (17·50) | 17 (14·17) | 4·14 | 0·387 |
| 40-57 | 123 (69·89) | 26 (14·77) | 27 (15·34) |  |  |
| ≥ 58 | 75 (78·95) | 12 (12·63) | 8 (8·42) |  |  |
| **Sex** |  |  |  |  |  |
| Male | 91 (71·09) | 19 (14·84) | 18 (14·06) | 0·09 | 0·952 |
| Female | 189 (71·86) | 40 (15·21) | 34 (12·93) |  |  |
| **Marital Status** |  |  |  |  |  |
| Unmarried | 32 (66·67) | 7 (14·58) | 9 (18·75) | 1·42 | 0·492 |
| Married | 248 (72·30) | 52 (15·16) | 43 (12·54) |  |  |
| **Religion** |  |  |  |  |  |
| Hindu | 254 (71·35) | 53 (14·89) | 49 (13·76) | 0·79 | 0·673 (F) |
| Non-Hindu | 26 (74·29) | 6 (17·14) | 3 (8·57) |  |  |
| **Ethnicity** |  |  |  |  |  |
| Janajati | 162 (75·35) | 28 (13·02) | 25 (11·63) | 3·28 | 0·193 |
| Others | 118 (67·05) | 31 (17·61) | 27 (15·34) |  |  |

**F = Fisher’s Exact Test.**

**Association between time spent with doctor and family size, language and area of residence**

| **Characteristics** | **Time Spent with Doctor** | | | **Chi-**  **square value** | **p-value** |
| --- | --- | --- | --- | --- | --- |
|  | **Satisfied(%)** | **Neutral (%)** | **Not Satisfied (%)** |  |  |
| **Native Language** |  |  |  |  |  |
| Nepali | 124 (67·76) | 31 (16·94) | 28 (15·30) | 2·52 | 0·282 |
| Non-Nepali | 156 (75·00) | 28 (13·46) | 24 (11·54) |  |  |
| **Area of Residence** |  |  |  |  |  |
| Rural/Urban Municipality | 244 (71·76) | 49 (14·41) | 47 (13·82) | 1·34 | 0·511 |
| Sub-metropolitan/Metropolitan | 36 (70·59) | 10 (19·61) | 5 (9·80) |  |  |
| **Family Size** |  |  |  |  |  |
| ≤5 | 192 (70·59) | 37 (13·60) | 43 (15·81) | 5·67 | 0·059 |
| >5 | 88 (73·95) | 22 (18·49) | 9 (7·56) |  |  |

**Association between time spent with doctor and education and economy related characteristics**

| **Characteristics** | **Time Spent with Doctor** | | **Chi-**  **square value** | **p-value** |
| --- | --- | --- | --- | --- |
|  | **Satisfied(%)** | **Not Satisfied (%)** |  |  |
| **Educational Status** |  |  |  |  |
| Illiterate | 62 (72·94) | 16 (18·82) | 7 (8·24) | 0·210 |
| Literate | 218 (71·24) | 43 (14·05) | 45 (14·71) |  |
| **Income*** |  |  |  |  |
| Present | 27 (58·70) | 6 (13·04) | 13 (28·26) | 0·006 |
| Absent | 253 (73·33) | 53 (15·36) | 39 (11·30) |  |
| **Occupation** |  |  |  |  |
| Homemaker | 107 (76·43) | 16 (11·43) | 17 (12·14) | 0·124 |
| Unemployed | 74 (76·29) | 11 (11·34) | 12 (12·37) |  |
| Others | 99 (64·71) | 31 (20·26) | 23 (15·03) |  |

***significantly associated (< 0.05).**

**Association between time spent with doctor and individual variables**

| **Characteristics** |  | | **Time Spent with Doctor** | | **Chi-square value** | **p-value** |
| --- | --- | --- | --- | --- | --- | --- |
|  | **Satisfied (%)** | **Neutral (%)** | | **Not Satisfied (%)** |  |  |
| **Time taken to reach health facility (in minutes)** | | | | | |  |
| <30 | 153 (70·18) | 33 (15·14) | | 32 (14·68) | 1·63 | 0·441 |
| 30-60 | 98 (71·53) | 22 (16·06) | | 17 (12·41) |  |  |
| >60 | 29 (80·56) | 4 (11·11) | | 3 (8·33) |  |  |
| **Self-reported Health Status** | | | |  |  |  |
| Good | 249 (73·67) | 48 (14·20) | | 41 (12·13) | 5·62 | 0·060 |
| Bad | 31 (58·49) | 11 (20·75) | | 11 (20·75) |  |  |
| **Type of Illness** |  |  | |  |  |  |
| Acute | 75 (72·82) | 12 (11·65) | | 16 (15·53) | 0·10 | 0·752 |
| Chronic | 205 (71·18) | 47 (16·32) | | 36 (12·50) |  |  |

**Association between time spent with doctor and insurance related characteristics**

| **Characteristics** | **Time Spent with Doctor** | | | **Chi-square value** | **p-value** |
| --- | --- | --- | --- | --- | --- |
|  | **Satisfied (%)** | **Neutral (%)** | **Not Satisfied (%)** |  |  |
| **Health Insurance Premium Affordability** | | | |  |  |
| Affordable | 274 (71·54) | 58 (15·14) | 51 (13·32) | 0·05 | 0·974 (F) |
| Not Affordable | 6 (75·00) | 1 (12·50) | 1 (12·50) |  |  |
| **Benefit from Insurance** |  |  |  |  |  |
| Yes | 276 (72·06) | 55 (14·36) | 52 (13·58) | 8·22 | 0·016 (F) |
| No | 4 (50·00) | 4 (50·00) | 0 (0·00) |  |  |
| **Willingness to Pay for higher insurance ceiling and better-quality service package** | | | | | |
| Yes | 128 (68·45) | 30 (16·04) | 29 (15·51) | 2·16 | 0·339 |
| No | 152 (74·88) | 28 (13·79) | 23 (11·33) |  |  |
| **WTP (in NPR)** |  |  |  |  |  |
| 4000-4500 | 83 (70·34) | 18 (15·25) | 17 (14·41) | 0·64 | 0·725 |
| ≥5000 | 44 (64·71) | 12 (17·65) | 12 (17·65) |  |  |
| **Type of Insurance** |  |  |  |  |  |
| Subsidized | 23 (85·19) | 4 (14·81) | 0 (0·00) | 4·61 | 0·100 (F) |
| Not Subsidized | 257 (70·60) | 55 (15·11) | 52 (14·29) |  |  |
| **Years of enrollment (in years)*** | |  |  |  |  |
| <3 | 91 (64·08) | 27 (19·01) | 24 (16·90) | 6·21 | 0·045 |
| ≥3 | 189 (75·90) | 32 (12·85) | 28 (11·24) |  |  |
| **Renewed insurance every year** | |  |  |  |  |
| Yes | 253 (71·47) | 2 (13·33) | 0 (0·00) | 2·52 | 0·284 (F) |
| No | 82 (71·93) | 13 (11·40) | 19 (16·67) |  |  |
| **Availability of medicines** |  |  |  |  |  |
| Available | 198 (71·48) | 46 (16·61) | 33 (11·91) | 2·82 | 0·244 |
| Unavailable | 82 (71·93) | 13 (11·40) | 19 (16·67) |  |  |
| **Knowledge of NHIP** |  |  |  |  |  |
| Adequate | 90 (71·43) | 17 (13·49) | 19 (15·08) | 0·76 | 0·684 |
| Inadequate | 190 (71·70) | 42 (15·85) | 33 (12·45) |  |  |

**F = Fisher’s Exact Test. WTP = Willingness to Pay. NPR = Nepalese Rupees. NHIP = National Health Insurance Program. *significantly associated (< 0.05).**

**Association between accessibility and convenience and socio-demographic characteristics**

| **Characteristics** | **Accessibility and Convenience** | | | **Chi-square value** | **p-value** |
| --- | --- | --- | --- | --- | --- |
|  | **Satisfied (%)** | **Neutral (%)** | **Not Satisfied (%)** |  |  |
| **Age** |  |  |  |  |  |
| <40 | 35 (29·17) | 45 (37·50) | 40 (33·33) | 5·64 | 0·228 |
| 40-57 | 52 (29·55) | 74 (42·05) | 50 (28·41) |  |  |
| ≥ 58 | 39 (41·05) | 30 (31·58) | 26 (27·37) |  |  |
| **Sex** |  |  |  |  |  |
| Male | 39 (41·05) | 30 (31·58) | 26 (27·37) | 4·16 | 0·125 |
| Female | 50 (39·06) | 45 (35·16) | 33 (25·78) |  |  |
| **Marital Status** |  |  |  |  |  |
| Unmarried | 18 (37·50) | 17 (35·42) | 13 (27·08) | 0·70 | 0·705 |
| Married | 108 (31·49) | 132 (38·48) | 103 (30·03) |  |  |
| **Religion** |  |  |  |  |  |
| Hindu | 116 (32·58) | 135 (37·92) | 105 (29·49) | 0·24 | 0·889 |
| Non-Hindu | 10 (28·57) | 14 (40·00) | 11 (31·43) |  |  |
| **Ethnicity*** |  |  |  |  |  |
| Janajati | 67 (31·16) | 93 (43·26) | 55 (25·58) | 6·18 | 0·046 |
| Others | 59 (33·52) | 56 (31·82) | 61 (34·66) |  |  |

***significantly associated (< 0.05)**

**Association between accessibility and convenience and family size, language and area of residence**

| **Characteristics** | **Accessibility and Convenience** | | | **Chi-square value** | **p-value** |
| --- | --- | --- | --- | --- | --- |
|  | **Satisfied (%)** | **Neutral (%)** | **Not Satisfied (%)** |  |  |
| **Native Language*** |  |  |  |  |  |
| Nepali | 60 (32·79) | 58 (31·69) | 65 (35·52) | 7·72 | 0·021 |
| Non-Nepali | 66 (31·73) | 91 (43·75) | 51 (24·52) |  |  |
| **Area of Residence** |  |  |  |  |  |
| Rural/Urban Municipality | 107 (31·37) | 134 (39·41) | 99 (29·12) | 1·89 | 0·389 |
| Sub-metropolitan/Metropolitan | 19 (37·25) | 15 (29·41) | 17 (33·33) |  |  |
| **Family Size** |  |  |  |  |  |
| ≤5 | 19 (37·25) | 15 (29·41) | 17 (33·33) | 3·7 | 0·157 |
| >5 | 44 (36·97) | 37 (31·09) | 38 (31·93) |  |  |

***significantly associated (< 0.05)**

**Association between accessibility and convenience and education and economy related characteristics**

| **Characteristics** | **Accessibility and Convenience** | | | **Chi-square value** | **p-value** |
| --- | --- | --- | --- | --- | --- |
|  | **Satisfied (%)** | **Neutral (%)** | **Not Satisfied (%)** |  |  |
| **Educational Status** |  |  |  |  |  |
| Illiterate | 27 (31·76) | 31 (36·47) | 27 (31·76) | 0·25 | 0·885 |
| Literate | 99 (32·35) | 118 (38·56) | 89 (29·08) |  |  |
| **Income*** |  |  |  |  |  |
| Present | 12 (26·09) | 25 (54·35) | 9 (19·57) | 6·01 | 0·050 |
| Absent | 114 (33·04) | 124 (35·94) | 107 (31·01) |  |  |
| **Occupation** |  |  |  |  |  |
| Homemaker | 47 (33·57) | 50 (35·71) | 43 (30·71) | 1·63 | 0·802 |
| Unemployed | 31 (31·96) | 35 (36·08) | 31 (31·96) |  |  |
| Others | 48 (31·37) | 64 (41·83) | 41 (26·80) |  |  |

***significantly associated (< 0.05).**

**Association between accessibility and convenience and hospital and health related characteristics**

| **Characteristics** | **Accessibility and Convenience** | | | **Chi-square value** | **p-value** |
| --- | --- | --- | --- | --- | --- |
|  | **Satisfied (%)** | **Neutral (%)** | **Not Satisfied (%)** |  |  |
| **Time taken to reach health facility (in minutes)** | | | |  |  |
| <30 | 76 (34·86) | 83 (38·07) | 59 (27·06) | 8·49 | 0·075 |
| 30-60 | 45 (32·85) | 52 (37·96) | 40 (29·20) |  |  |
| >60 | 5 (13·89) | 14 (38·89) | 17 (47·22) |  |  |
| **Self-reported Health Status** | |  |  |  |  |
| Good | 110 (32·54) | 129 (38·17) | 99 (29·29) | 0·20 | 0·904 |
| Bad | 16 (30·19) | 20 (37·74) | 17 (32·08) |  |  |
| **Type of Illness*** |  |  |  |  |  |
| Acute | 29 (28·16) | 31 (30·10) | 43 (41·75) | 9·95 | 0·007 |
| Chronic | 97 (33·68) | 118 (40·97) | 73 (25·35) |  |  |

***significantly associated (< 0.05).**

**Association between accessibility and convenience and insurance related characteristics**

| **Characteristics** | **Accessibility and Convenience** | | | **Chi-square value** | **p-value** |
| --- | --- | --- | --- | --- | --- |
|  | **Satisfied (%)** | **Neutral (%)** | **Not Satisfied (%)** |  |  |
| **Health Insurance Premium Affordability** | | | |  |  |
| Affordable | 125 (32·64) | 147 (38·38) | 111 (28·98) | 4·32 | 0·115 (F) |
| Not Affordable | 1 (12·50) | 2 (25·00) | 5 (62·50) |  |  |
| **Benefit from Insurance** | |  |  |  |  |
| Yes | 126 (32·90) | 149 (38·90) | 108 (28·20) | 19·36 | <0·001 (F) |
| No | 0 (0·00) | 0 (0·00) | 8 (100·00) |  |  |
| **Willingness to Pay for higher insurance ceiling and better-quality service package** | | | | | |
| Yes | 66 (35·29) | 76 (40·64) | 45 (24·06) | 5·13 | 0·077 |
| No | 60 (29·56) | 73 (35·96) | 70 (34·48) |  |  |
| **WTP (in NRP)** |  |  |  |  |  |
| 4000-4500 | 36 (30·51) | 49 (41·53) | 33 (27·97) | 3·75 | 0·153 |
| ≥5000 | 29 (42·65) | 27 (39·71) | 12 (17·65) |  |  |
| **Type of Insurance** | |  |  |  |  |
| Subsidized | 10 (37·04) | 9 (33·33) | 8 (29·63) | 0·38 | 0·826 |
| Not Subsidized | 116 (31·87) | 140 (38·46) | 108 (29·67) |  |  |
| **Years of enrollment (in years)** | |  |  |  |  |
| <3 | 43 (30·28) | 51 (35·92) | 48 (33·80) | 1·83 | 0·401 |
| ≥3 | 83 (33·33) | 98 (39·36) | 68 (27·31) |  |  |
| **Renewed insurance every year** | |  |  |  |  |
| Yes | 116 (32·77) | 136 (38·42) | 102 (28·81) | 3·26 | 0·196 (F) |
| No | 2 (13·33) | 6 (40·00) | 7 (46·67) |  |  |
| **Availability of medicines*** | |  |  |  |  |
| Available | 100 (36·10) | 106 (38·27) | 71 (25·63) | 9·65 | 0·008 |
| Unavailable | 26 (22·81) | 43 (37·72) | 45 (39·47) |  |  |
| **Knowledge of NHIP*** | |  |  |  |  |
| Adequate | 50 (39·68) | 47 (37·30) | 29 (23·02) | 6·01 | 0·049 |
| Inadequate | 76 (28·68) | 102 (38·49) | 87 (32·83) |  |  |

**F = Fisher’s Exact Test. WTP = Willingness to Pay. NPR = Nepalese Rupees. NHIP = National Health Insurance Program.**

***significantly associated (< 0.05).**
