# Supplementary material for: Patient satisfaction among national health insurance enrollees in an accredited hospital of Kathmandu Valley: A cross-sectional, mixed methods study
Source: PLoS One. 2026 Mar 20;21(3):e0345353. doi: 10.1371/journal.pone.0345353 (PMC13004337; doi:10.1371/journal.pone.0345353)
Supplement: S4 Table — Codebook for detailed qualitative findings. (DOCX) [file pone.0345353.s005.docx]

**S4 Table. Codebook.** Codebook for detailed qualitative findings.

| **Theme** | **Code** | **Code Definition/ Description** | **Examples** | **Key Quotes** | **References** |
| --- | --- | --- | --- | --- | --- |
| Issues with medicine quality and availability | Poor quality medicines | Discussions of medicines that are deemed to be poor quality or those that lack sufficient drug efficacy. | "They are often met with reviews of being inferior and poor quality with lack of drug efficacy. " - F01 "There are also issues with the quality of medicines. If we write generic medicine names, we are left unsure of what medicines the patients actually get. There is a difference in brands of medicines and their efficacy and if it works for the patients or not. It is not possible for us to regulate the medicines." - M05 "there is no consistency of the quality of the medicines. Sometimes, we receive great quality medicines while other times we receive poor quality medicines. The medicines also get exchanged or an alternative medicine of the same name is sent to us." - M06 |  | M05, M06, F01 |

| **Theme** | **Code** | **Code Definition/ Description** | **Examples** | **Key Quotes** | **References** |
| --- | --- | --- | --- | --- | --- |
| Issues with medicine quality and availability | Medicine unavailable | Discussions of how medicines that are listed under the NHIP remain unavailable | "Many medications that are listed under the insurance program are not frequently available." - F02 "The insurance medicines are also not enough in all cases. When we run out of the insurance medicine stock, the patients get disappointed when they have to wait for hours in line and they cannot even get the medicines they need." - F07 | “They [the patients] are satisfied because they receive medicines free of cost under the program and do not have to pay out of their pockets.” - M06 | F02, F07, M06 |
|  | Medicine prescription policies | Discussions of medicine prescription policies that renders difficulty to patients with chronic illnesses who require medicines every month. Indicates that medicine prescription papers work only for a single day and patients are forced to get another prescription if they were to take medicines a day after consultation | "They also have to come every month for medications when there should be provisions of at least 3 month medicine prescription for patients with chronic and non-communicable diseases. It is a waste of their times to come back every month and every other day and we are also burdened due to the same thing." - M05 |  | M05 |

| **Theme** | **Code** | **Code Definition/ Description** | **Examples** | **Key Quotes** | **References** |
| --- | --- | --- | --- | --- | --- |
| Moral Hazard | Service abuse by the private accredited facilities | Discussions of how services covered by NHIP benefit package is misued by private entities who are accredited under the NHIP | "The private health facilities put in a lot of unnecessary investigations considering they will get reimbursed a lot. they have extended the insurance services to private for the universal coverage, but a lot of issues are arising due to this. There is situation of how they have been including tests that are not included under the insurance services. " F07 |  | F07 |
|  | Demand for insurance medicine prescription | Discussion of how insured patients sometimes pressure medical consultants to prescribe them with medication even after physician did not indicate any. | “There is a lot of cases of unnecessary consultations and medications that the patients come here for when they wouldn't have if they were not insured. There are a lot of investigations that are also unnecessary. At this rate, the program might fail or collapse." - F02 | "There is no point in doctor consultations if patients only come here to get medications. There is a huge demand of patients to get medicines even if they do not require them. Most patients want more medications to be added in their prescription paper." - M05 |  |

| **Theme** | **Code** | **Code Definition/ Description** | **Examples** | **Key Quotes** | | **References** |
| --- | --- | --- | --- | --- | --- | --- |
| Moral Hazard | Demand for insurance medicine prescription | Discussion of how insured patients sometimes pressure medical consultants to prescribe them with medication even after physician did not indicate any. | "The users do not have the understanding of how insurance system works and they are only catered that they will get health services free of cost once they are insured. There has been a lot of misuse. People are using it extensively while the genuine needy patients are not been able to make most out of it. It is a double-edged sword in my opinion." - M05  "And if it has been close to a year, they come for unnecessary general tests. Such practices of looking at how much amount is remaining and visiting here for the sole purpose of spending the money. They come for general tests even if they do not have any kinds of ailments. So, there has been unnecessary misuse of the insurance system. So, there is a lack of awareness among the service users and they think that they need to claim all the services that are given to them. The patients who actually have disease and require investigations and treatment swiftly are disrupted due to these kinds of practices that lead to more crowds. " - F07 | | "More than 70-80% patients are genuine patients. Another 25-30% percentage of patient misuse and come for the sake of getting unnecessary consultations, tests and unnecessary medications. Sometimes they fake their symptoms just for the sake of getting the medicines. We counsel and try to persuade them to not take unnecessary medicines but if the patient persists, we are compelled to give them medications. For that, if the insurance board provided us some directions as to tackling this kind of situation and we had the authority to cancel their medications. the misuse could be minimized." - M04 "I think I want to appeal that the patients should only take the insurance services if it's necessary to. If they take services without the real need or necessity, others are also getting affected as we know that insurance is a collective fund. If the fund is misused, needy persons will not sufficiently get the services they require. Awareness should be made about this so that misuse of the fund is minimized." - M04 | M05, F02, M04 |
| **Theme** | **Code** | **Code Definition/ Description** | **Examples** | | **Key Quotes** | **References** |
| Moral Hazard | Need for co-payment mechanism | Discussion of how there is need to establish a co-payment by the insured to control the unnecessary consultation and misuse of healthcare services under health insurance benefit package. | "There should be a mechanism of copayment where the insured should be responsible to pay certain percentage of the total amount, say 20% and 80% gets covered by the insurance. This will greatly reduce unnecessary consultations and check-ups and also lower the patient inflow." - F02 "There should also be only about 70% or more than 50% coverage done by the insurance and the rest should be made to be paid by the insured patients themselves." - M06 | |  | F02, M06 |
| Reimbursement issues | Reimburse-ment delays and discrepanci-es | Discussion of how reimbursement to the hospital is delayed and cuts being made on the claim amount due to technical issues | "The reimbursement is not done in a timely manner and also the exact reimbursement amount proposed is not received. There are some cuts made in the insurance claim amount. On top of that, health service providers who have to face the burden of high patient inflow are not incentivized in any way. We do not have a fee for services or any mechanism to incentivize the health service providers" - M03 | |  | M03 |

| **Theme** | **Code** | **Code Definition/ Description** | **Examples** | **Key Quotes** | **References** |
| --- | --- | --- | --- | --- | --- |
| Reimbursement issues | Technical software issues | Discussion about how servers are sometimes down which hinders the hospital to claim amount on time. | "Other problems are about the server, there is also conditions of the server being down, and we cannot claim on time due to that. That is something that is a problem with the system, even now it is still pending. " - F07 |  | F07 |
|  | Revision of reimbursement rates for in-patient medical procedures and services expansion | Discussion of how scope of health services under benefit package needs to be expanded to accommodate investigation tests and changes in reimbursement charges fixed for some medical in-patient. | "There is lack of inclusion of some important medicines under the insurance listed medicines other than the basic medicines. Also, incorporation of further investigation tests like Vitamin D tests, dental checkups, hormonal tests and other vitamin blood tests [under insurance benefit package] can also help improve the insurance program." - M04 | "There is a need to review and make changes to the OT (operation theatre) reimbursement charges. Some simple procedures are heavily charged whereas complex procedures are lightly charged." - M06 | M04, M06 |
|  | Short duration for reimbursement claim | Discussion of how period to claim reimbursement is limited to one-week, considered problematic and in-sufficient. | "…we have to claim insurance amount every single day. However, if we cannot claim for up to one week, such reimbursement can no longer be applicable and it gets nullified. If one patient gets admitted and discharged, their claim should be done within a week" - F07 |  | F07 |

| **Theme** | **Code** | **Code Definition/ Description** | **Examples** | **Key Quotes** | **References** |
| --- | --- | --- | --- | --- | --- |
| Reimbursement issues | Proxy patients for geriatric guardians with chronic illnesses | Discussion of how patients who present to the health facility on the behalf of their elderly family members who require monthly medicines act as 'proxy patients' making it difficult to proceed with reimbursements. | "…the patients who have chronic diseases like HTN, diabetes who have to take medications long term, they have the same medicines to take. Some of them belong to the geriatric, who are 70 years or older who have to come time and again only to get the medicines. For them, their guardians come to take the medicines, so they are like a proxy here. And we cannot give the proxy patients the medicines. So, that kind of problem exists. If we do not give them the medicines, the old people have a hard time, and if we do give the proxy patients medicines, it is difficult to show in the reimbursement system." - F07 |  | F07 |

| **Theme** | **Code** | **Code Definition/ Description** | **Examples** | **Key Quotes** | **References** |
| --- | --- | --- | --- | --- | --- |
| De-incentivization or demotivation among the service providers | Heavy work burden and extended working hours | Discussion regarding higher assignment of OPD duty due to high patient inflow resulting in working hours getting extending by an hour or two past the designated working hours. | "There could be quota system as to how many patients we are required to attend in a day and we could pace ourselves according to that and provide good quality services." - M05 | "Personally, I think this mechanism of insurance program for service delivery is effective and beneficial for patients. However, for us at the end of supply side, we have to bear the burden of high patient inflow. The hospital patient load has doubled/tripled but the human resources present are in the same amount which creates overburden to the entire healthcare system" - M03 "There should be some type of mandate as to how many patients a doctor has to attend in a day or so. There should be an exact number of patients that the doctors should serve each day so that it does not burden the service providers. This will help improve the quality of health service." - M03 | M05, M03 |
|  | Lack of capitation or fees | Discussion of how service providers do not get capitation or fee for services for attending to insured patients. | "We do not get the insurance reimbursement ourselves. It is handled by the administration. We only take our basic salary, which is the government set salary for 9th level medical consultants." - F01 |  | F01 |

| **Theme** | **Code** | **Code Definition/ Description** | **Examples** | **Key Quotes** | **References** |
| --- | --- | --- | --- | --- | --- |
| De-incentivization or demotivation among the service providers | Tedious referral system | Discussion regarding the need to obtain a referral document every time patients follow-up at the referred health institution which places burden on both patients and healthcare providers. | ". The referral process is time consuming both for us and the patients. In some cases when we refer patients to a hospital, say ABC Hospital, and it doesn't have the services required, they have to come back to us to get referred to say XYZ hospital. There is limited time for the referral documents to remain valid, which is only one week." - F02 "The referral system could also be direct at the concerned hospitals so that they will not be need lengthy paperwork. It could be digitalized." - M03 | " If the services for chronic diseases and diseases like cancer is not available in this hospital, there is a provision that they do not have to come repeatedly for referral and they can get services for their treatment by getting one time referral. However, after the problem kept happening, in the second time, the problem has been occurring repeatedly, so this time they have issued a formal notice. It is uncertain how effective this notice will be and it will depend on how the health facilities will take this notice and how they will implement it. " - F07 | F07, M03, F02 |

| **Theme** | **Code** | **Code Definition/ Description** | **Examples** | **Key Quotes** | **References** |
| --- | --- | --- | --- | --- | --- |
| De-incentivization or demotivation among the service providers | Limited human resource availability | Discussion of the absence of appropriate number of consultants to attend to the regular patient inflow | "The hospital is severely understaffed. We only have two of us working in the XXX department and also have to do consultations at the ward. We have to attend about 60 patients on a daily basis. Sometimes, the number goes up to 100 patients in a single day. This adds to the work burden." - F02 "There is less doctor patients or nurse patient ratio. So, HR is still deficit as mentioned earlier. Due to that, patient are not given enough time. So, all their queries may not be answered. However, they are given services and time necessary to them in my opinion. They do have the expectation that they prefer longer time for consultations. That expectation may or may not have been met in that aspect." - F07 | "Hospitals accredited for insurance services should also be checked if they have enough human resources to attend the insured patients. If there are only 2-3 physicians in a hospital and 200-300 patients are sent to that hospital, the patients cannot get good quality health care. They are only concerned about the quantity and the compromise made in the quality is overlooked. Allocation of patients should be according to the HR available in the accredited facilities." - M05 "There is increased patient inflow. However, the doctor to patient ratio does not match up to the patient inflow. There is need of a proper management in that regards." - M6 | F07, F02, M06, M05 |

| **Theme** | **Code** | **Code Definition/ Description** | **Examples** | **Key Quotes** | **References** |
| --- | --- | --- | --- | --- | --- |
| Lack of coordinated roll-out of health insurance services | Misalignment of hospital resource capacity and flow of insured patients | Discussion of how the insurance program failed to take hospital resources and capacity under consideration before designating it as a primary contact point for receiving health insurance services. | "The government has the perception that everyone should be insured under the national health insurance program and get services accordingly, but the human resource according to that is very insufficient. There is also a lack of infrastructure. The insurance board has not taken this under consideration, they have only thought about giving out services, but the availability of human resources or infrastructures, and also the software were not considered. There is problem of work duplication." - F07 ""The outpatient inflow especially after the health insurance program has increased a lot. This has not been taken into consideration with respect to the capacity of the hospital and the health workers currently available. "- F01 | "There is need for developments in infrastructures and fulfilling HR deficit from the policy level. For this, not only the hospital and the health providers, the whole team should come together, like the health department and the health ministry should be coordinated." - F07 | F01, F07 |

| **Theme** | **Code** | **Code Definition/ Description** | **Examples** | **Key Quotes** | **References** |
| --- | --- | --- | --- | --- | --- |
| Lack of coordinated roll-out of health insurance services | Scare training programs organized by NHIP | Discussion of how there are rarely any trainings or refresher training provided on how to run the health insurance program and need for such trainings for health professionals. | "The insurance staff working here can be sent to training regarding how to claim for 2-4 days by coordinating with the insurance board. I do not have clear information on this. The hospital will most likely need to take an initiative on this." - F07 | "There is need of provisions of trainings and refresher trainings thereafter. That way, the service providers can also easily provide services and the insured can swiftly receive those services." - F7 | F07 |
|  | Limited monitoring visits from HIB | Discussion of how the health insurance board needs to initiate active coordination with accredited public health facilities and run monitoring and evaluation visits to improve services provided. | "…the insurance board can play a role in coordinating, and take action when referrals are frequently asked even when the notices for one time referral requirements are issued about what issues are leading to this. There should be two-way communication done by them in this regard. So, if monitoring and evaluation is done in such cases, patients can be satisfied and we can also be motivated to provide services properly. " - F07 |  | F07 |
|  | Perceived lack of incorporation of health provider's voice and interests in the insurance scheme | Discussion of how perspectives and feedback from health providers are not considered or included in design the national health insurance program. | "There is a lack of input form the health service providers’ side and their concerns are not well-considered when designing the program. There is no motivation for the health service provider considering the high patient inflow of patients after the initiation of the health insurance program. " - F01 |  | F01 |
| **Theme** | **Code** | **Code Definition/ Description** | **Examples** | **Key Quotes** | **References** |
| Time spent with doctors | Short consultation during rush hours | Discussions of how the health providers are forced to shorten the consultation times to accommodate high patient inflow. | "We are required to give about 5 minutes in each consultation with our patients. However, practically that is not always possible. At least one doctor has to be available for 24-hour duty. There is a schedule for who will be responsible for emergency calls. Our usual work can extend from 8 am up to 5 or 6 pm in the evening."- F01 "There is less doctor patients or nurse patient ratio. So, HR is still deficit as mentioned earlier. Due to that, patients are not given enough time. So, all their queries may not be answered. However, they are given services and time necessary to them in my opinion. They do have the expectation that they prefer longer time for consultations. That expectation may or may not have been met in that aspect." - F07 | "The health service that we cater right now is more quantity-based than quality based and we need to attend to every patient regardless of the time they are getting for the consultation." - F01 | F07, F01 |
|  | Mixed reactions from patients due to provider's assertiveness | Discussion of how patients might perceive assertiveness from health providers negatively that might affect their satisfaction with health services. |  | "On rush hours, we have to be more assertive which sometimes they do not take well and might not like it." - F01 | F01 |
