# Supplementary material for: Patient satisfaction among national health insurance enrollees in an accredited hospital of Kathmandu Valley: A cross-sectional, mixed methods study
Source: PLoS One. 2026 Mar 20;21(3):e0345353. doi: 10.1371/journal.pone.0345353 (PMC13004337; doi:10.1371/journal.pone.0345353)
Supplement: S5 Table — A joint table analysis of quantitative and follow-up qualitative findings regarding insurance-related characteristics and patient satisfaction. (DOCX) [file pone.0345353.s006.docx]

**S5 Table. Joint Table.** A joint table analysis of quantitative and follow-up qualitative findings regarding insurance-related characteristics and patient satisfaction**.**

| **Topics of integrative analysis** | **Quantitative findings** | **Qualitative findings** | **Inferences** |
| --- | --- | --- | --- |
|  | **Frequency (%)** |  |  |
| 1. **Hospital and insurance-related characteristics** | | | |
| Years of enrollment: ≥ 3 | 63.68 | - Healthcare providers having good interpersonal relationship with older patients | Frequent contacts and consultation visits of older patients to healthcare providers improved relationship with patients. |
| Availability of medicines | 70.84 | - Frequent stock-outs in hospital of insurance-listed medicines - Receiving medicines (free of cost) was crucial to patient satisfaction - Reliance on private pharmacies | Availability of insurance-listed medications influenced patient satisfaction which was determined by coordinated supply of medicines |
| Willingness to Pay (WTP) | 47.95 | - Need of co-payment to curb moral hazard practices | Willingness to pay for better insurance services can be complemented with the need of co-payment mechanism in order to limit moral hazard practices |

| **Topics of integrative analysis** | **Quantitative findings** | **Qualitative findings** | **Inferences** |
| --- | --- | --- | --- |
|  | **Frequency (%)** |  |  |
| **B. Satisfaction Domain** | | | |
| Time spent with doctor | 71.61 | - Good relationship of healthcare providers with older patients who visited them for a long period of time - Healthcare providers not being able to provide enough time for consultation due to high patient inflow - Limited interaction with patients on their consultation visits | Satisfaction in the domain of time spent with doctor is influenced directly by the long period of visitations made by older patients as well as the consultation time |
| General satisfaction | 36.06 | - Dissatisfaction among patients stem from medicine unavailability, lack of medical specialists as well as high inflow of insured patients disrupting regular out-patient services | Lower general patient satisfaction can be explained by stock-outs of insurance listed medicines, lack of specialists and high patient inflow |
| Accessibility and convenience | 32.33 | - High patient inflow - Increased work burden among service providers - Lack of specialist services with only medical consultants available in OPD | Unavailability of medical specialist services in OPD explains the lower satisfaction in the domain of accessibility and convenience |

| **Topics of integrative analysis** | **Quantitative findings** | **Qualitative findings** | **Inferences** |
| --- | --- | --- | --- |
|  | **Frequency (%)** |  |  |
| **C. Agreement statements** | | | |
| I think my doctor’s office has everything needed to provide complete medical care. | 89.50 | - Lack of infrastructure and equipment in hospital | Basic consultation services are available whereas specialist services require medical equipment that are not available in the OPD |
| I feel confident that I can get the medical care I need without being set back financially. | 65.50 | - Having to buy medicines from pharmacy during stock-out periods | Out-of-pocket expenditure when buying medicines from pharmacies stands as one of the major hurdles in receiving medical care without having financial constraints |
| My doctors treat me in a very friendly and courteous manner | 82.60 | - Good relationship with older patients - Overall good interpersonal relationship of healthcare providers with patients | Higher agreement with friendly treatment by providers can be explained by good interpersonal relationship with patients, especially elderly. |
| Those who provide my medical care sometimes hurry too much when they treat me | 20.50 | - Mixed reactions from patients due to providers’ assertiveness during rush hours - Short consultation time due to high patient inflow | Providers’ assertion during rush hours might also have affected the satisfaction felt by patients. |
| I have easy access to the medical specialists I need | 36.10 | - High patient inflow - Lack of specialist services - Tedious referral processes | Lower agreement in access to specialist services was explained by lack of medical specialists in the OPD and difficulty in referral processes |

| **D. Predictors of patient satisfaction** | **Statistically significant (p-value)** | **Qualitative findings** | **Inferences** |
| --- | --- | --- | --- |
| Medicine availability | Significant (p<0.001) | - Patients satisfied when they receive medications (free of cost) after their treatment - Patients are disappointed when they do not receive medicines under the insurance program | Insurance-listed medicine availability surfaced as explanations for improved patient satisfaction |
| Adequate knowledge of the NHIP | Significant (p=0.009) | - Lack of knowledge of the insurance risk pooling mechanism leading to misuse and unnecessary demands of medicine prescriptions - Lack of knowledge and negligence by the insured patients leading to missed reimbursement dates | Knowledge of insurance-related provisions along with risk pooling functions determines conscious use of insurance services which in turn describes patient satisfaction |
| Type of illness: Chronic | Significant (p=0.026) | - Patients with chronic illnesses have frequent consultation visits for medicine prescription - Good interpersonal relationship with older patients | Frequent consultation visits after every three months and availability of medicines for major chronic illnesses presented as the explanation for higher odds of satisfaction among patients with chronic illnesses |
