# Supplementary material for: Patient satisfaction among national health insurance enrollees in an accredited hospital of Kathmandu Valley: A cross-sectional, mixed methods study
Source: PLoS One. 2026 Mar 20;21(3):e0345353. doi: 10.1371/journal.pone.0345353 (PMC13004337; doi:10.1371/journal.pone.0345353)
Supplement: S6 Table — The full table showing analysis in detailed form. (DOCX) [file pone.0345353.s007.docx]

**S6 Table. Ordinal Logistic Regression Analysis.** The full table showing analysis in detailed form.

| **Satisfaction Domain** | **Characteristics** | **Reference category = ‘Not Satisfied’** | | | | | | | |
| --- | --- | --- | --- | --- | --- | --- | --- | --- | --- |
|  |  | **Satisfied** | | | | **Neutral** | | | |
|  |  | **COR (95% CI)** | **p-value** | **AOR (95% CI)** | **p-value** | **COR (95% CI)** | **p-value** | **AOR (95% CI)** | **p-value** |
| **General Satisfaction** | **Type of Illness** |  |  |  |  |  |  |  |  |
|  | Chronic | 1.22 [0.74-1.99] | 0.435 | - | - | 0.36 [0.15-0.87] | **0.023** | - | - |
|  | Acute | Ref |  | Ref |  | Ref |  | Ref |  |
| **Technical Quality** | **Age (in years)** |  |  |  |  |  |  |  |  |
|  | <40 | 0.86 [0.40-1.87] | 0.719 | 1.35 [0.64-3.39] | 0.516 | 0.85 [0.35-2.05] | 0.721 | 1.05 [0.38-2.88] | 0.719 |
|  | 40-57 | 1.72 [0.77-3.87] | 0.187 | 1.73 [0.73-4.11] | 0.214 | 2.67 [1.12-6.51] | 0.028 | 2.38 [0.95-5.97] | 0.187 |
|  | ≥ 58 | Ref |  | Ref |  |  |  |  |  |
|  | **Marital Status** |  |  |  |  |  |  |  |  |
|  | Married | 3.38 [1.59-7.14] | 0.001 | 2.14 [0.88-5.20] | 0.095 | 4.76 [1.89-11.97] | 0.001 | 3.41 [1.20-9.66] | 0.021 |
|  | Unmarried | Ref |  | Ref |  |  |  |  |  |
|  | **Religion** | | |  |  |  |  |  |  |
|  | Hindu | 3.34 [1.30-8.57] | 0.012 | 3.18 [1.04-9.79] | **0.043** | 1.41 [0.55-3.63] | 0.471 | 1.31 [-.42-4.14] | 0.643 |
|  | Non-hindu | Ref |  | Ref |  |  |  |  |  |
|  | **Ethnicity** |  |  |  |  |  |  |  |  |
|  | Janajati | 2.37 [1.26-4.49] | 0.008 | 1.68 [0.42-6.78] | 0.465 | 1.50 [0.75-2.9] | 0.247 | 2.92 [0.68-12.42] | 0.148 |
|  | Others | Ref |  | Ref | 0.146 |  |  |  |  |
|  | **Mother Language** | | |  |  |  |  |  |  |
|  | Non-nepali | 2.33 [1.23-4.40] | 0.009 | 1.33 [0.34-5.27] | 0.683 | 1.21 [0.61-2.41] | 0.585 | 0.44 90.10-1.85] | 0.261 |
|  | Nepali | Ref |  | Ref |  | Ref |  | Ref |  |
|  | **Occupation** |  |  |  |  |  |  |  |  |
|  | Homemakers | 2.63 [1.26-5.49] | 0.010 | 2.17 [0.96-4.90] | 0.063 | 2.25 [1.02-4.99] | 0.046 | 1.92 [0.79-4.55] | 0.146 |
|  | Unemployed | 3.21 [1.32-7.82] | 0.010 | 3.03 [1.15-7.98] | **0.025** | 2.64 [1.02-6.83] | 0.046 | 2.45[0.88-6.85] | 0.086 |
|  | Others | Ref |  | Ref |  | Ref |  | Ref |  |
|  | **Time taken to reach health facility (in mins.)** | | |  |  |  |  |  |  |
|  | <30 | 0.57 [0.13-2.58] | 0.465 | 0.40 [0.08-1.96] | 0.258 | 0.43 [0.08-2.10] | 0.297 | 0.35 [0.06-1.88] | 0.222 |
|  | 30-60 | 0.23 [0.05-1.04] | 0.057 | 0.21 [0.04-1.03] | 0.055 | 0.38 [0.08-1.85] | 0.229 | 0.33 [0.06-1.76] | 0.195 |
|  | >60 | Ref |  | Ref |  | Ref |  | Ref |  |
|  | **Self-Reported Health Status** | | | |  |  |  |  |  |
|  | **Good** | 2.62 [1.21-5.65] | 0.014 | 2.71 [1.13-6.50] | **0.025** | 2.18 [0.93-5.09] | 0.073 | 2.85 [1.08-7.47] | 0.034 |
|  | **Bad** | Ref |  | Ref |  | Ref |  | Ref |  |
|  | **WTP** |  |  |  |  |  |  |  |  |
|  | **Yes** | 0.70 [0.38-1.31] | 0.269 | 1.22 [0.60-2.47] | 0.578 | 1.55 [0.78-3.05] | 0.210 | 0.56 [0.26-1.19] | 0.132 |
|  | **No** | Ref |  | Ref |  | Ref |  | Ref |  |
|  | **Years of Enrollment (in years)** | | |  |  |  |  |  |  |
|  | ≥3 | 2.02 [1.07-3.79] | 0.029 | 1.23 [0.60-2.53] | 0.567 | 1.29 [0.65-2.55] | 0.460 | 0.92 [0.43-1.97] | 0.820 |
|  | <3 | Ref |  | Ref |  | Ref |  | Ref |  |
| **Interpersonal Manner** | **Income** |  |  |  |  |  |  |  |  |
|  | Absent | 3.87 [1.38-10.79] | 0.010 | 3.87 [1.38-10.86] | **0.010** | 2.43 [0.74-7.98] | 0.144 | 2.43 [0.74-7.99] | 0.144 |
|  | Present | Ref |  | Ref |  | Ref |  | Ref |  |
|  | **Type of Illness** |  |  |  |  |  |  |  |  |
|  | Acute | 2.17 [0.85-5.52] | 0.103 | 2.17 [0.85-5.58] | 0.107 | 1.15 [0.41-3.25] | 0.790 | 1.15 [0.41-3.27] | 0.792 |
|  | Chronic | Ref |  | Ref |  | Ref |  | Ref |  |
| **Communication** | **Occupation** |  |  |  |  |  |  |  |  |
|  | Homemakers | 2.22 [0.74-6.64] | 0.153 | - | - | 1.83 [0.58-5.70] | 0.300 | - | - |
|  | Unemployed | 3.05 [0.82-11.31] | 0.096 | - | - | 1.17 [0.29-4.76] | 0.826 | - | - |
|  | Others | Ref |  | Ref |  | Ref |  | Ref |  |
| **Financial Aspect** | **Marital Status** |  |  |  |  |  |  |  |  |
|  | Married | 2.44 [1.16-5.12] | 0.018 | 2.14 [0.95-4.82] | 0.065 | 1.23 [0.55-2.79] | 0.614 | 1.34 [0.55-3.28] | 0.520 |
|  | Unmarried | Ref |  | Ref |  | Ref |  | Ref |  |
|  | **Religion** |  |  |  |  |  |  |  |  |
|  | Hindu | 1.36 [0.50-3.66] | 0.549 | 1.16 [0.39-3.37] | 0.785 | 0.43 [0.16-1.17] | 0.098 | 0.58 [0.19-1.72] | 0.328 |
|  | Non-hindu | Ref |  | Ref |  | Ref |  | Ref |  |
|  | **Ethnicity** |  |  |  |  |  |  |  |  |
|  | Janajati | 1.61 [0.95-2.72] | 0.079 | 1.55 [0.88-2.75] | 0.130 | 2.41 [1.28-4.55] | **0.007** | 2.79 [1.41-5.51] | **0.003** |
|  | Others | Ref |  | Ref |  | Ref |  | Ref |  |
|  | **Family size** |  |  |  |  |  |  |  |  |
|  | ≤5 | 0.93 [0.53-1.62] | 0.797 | 0.98 [0.54-1.76] | 0.932 | 2.01 [0.97-4.11] | 0.058 | 1.84 [0.87-3.89] | 0.110 |
|  | >5 | Ref |  | Ref |  | Ref |  | Ref |  |
|  | **Occupation** |  |  |  |  |  |  |  |  |
|  | Homemaker | 1.49 [0.82-2.75] | 0.191 | 1.18 [0.62-2.28] | 0.606 | 0.74 [0.36-1.51] | 0.41 | 0.72 [0.34-1.56] | 0.413 |
|  | Unemployed | 1.59 [0.81-3.12] | 0.181 | 1.44 [0.70-2.95] | 0.320 | 0.67 [0.29-1.52] | 0.34 | 0.61 [0.256-1.44] | 0.258 |
|  | Others | Ref |  | Ref |  | Ref |  | Ref |  |
|  | **Years of enrollment** | |  |  |  |  |  |  |  |
|  | ≥3 | 1.74 [1.01-2.99] | 0.045 | 1.48 [0.82-2.667] | 0.195 | 0.72 [0.39-1.34] | 0.303 | 0.59 [0.30-1.17] | 0.59 |
|  | <3 | Ref |  | Ref |  | Ref |  | Ref |  |
|  | **Availability of medicines** |  |  |  |  |  |  |  |  |
|  | Available | 4.10 [2.35-7.13] | <0.001 | 4.06 [2.30-7.17] | **<0.001** | 2.66 [1.39-5.10] | 0.003 | 2.83 [1.44-5.53] | **0.002** |
|  | Unavailable | Ref |  | Ref |  | Ref |  | Ref |  |
| **Time spent with doctor** | **Income** |  |  |  |  |  |  |  |  |
|  | Absent | 3.12 [1.48-6.56] | 0.003 | 3.19 [1.51-6.77] | **0.002** | 2.94 [1.03-8.43] | 0.044 | 2.95 [1.03-8.45] | **0.044** |
|  | Present | Ref |  | Ref |  | Ref |  | Ref |  |
|  | **Years of enrollment** | |  |  |  |  |  |  |  |
|  | ≥3 | 1.78 [0.98-3.24] | 0.059 | 1.83 [0.99-3.36] | 0.052 | 1.02 [0.48-2.15] | 0.967 | 1.04 [0.49-2.22] | 0.916 |
|  | <3 | Ref |  | Ref |  | Ref |  | Ref |  |
| **Accessibility and Convenience** | **Ethnicity** |  |  |  |  |  |  |  |  |
|  | Janajati | 1.26 [0.76-2.09] | 0.371 | 0.71 [0.23-2.16] | 0.547 | 1.84 [1.12-3.02] | 0.015 | 1.19 [0.41-3.44] | 0.756 |
|  | Others | Ref |  | Ref |  | Ref |  | Ref |  |
|  | **Mother Language** | |  |  |  |  |  |  |  |
|  | Non-Nepali | 1.40 [0.85-2.33] | 0.191 | 2.02 [0.66-6.15] | 0.215 | 2.00 [1.22-3.27] | 0.006 | 1.88 [0.65-5.44] | 0.242 |
|  | Nepali | Ref |  | Ref |  | Ref |  | Ref |  |
|  | **Income** |  |  |  |  |  |  |  |  |
|  | Absent | 0.79 [0.32-1.97] | 0.627 | 0.89 [0.35-2.32] | 0.820 | 0.42 [0.19-0.93] | 0.033 | 0.39 [0.16-0.91] | **0.028** |
|  | Present | Ref |  | Ref |  | Ref |  | Ref |  |
|  | **Type of Illness** |  |  |  |  |  |  |  |  |
|  | Chronic | 1.97 [1.13-3.45] | 0.018 | 1.92 [1.08-3.42] | **0.026** | 2.24 [1.29-3.87] | 0.004 | 2.36 [1.34-4.15] | **0.003** |
|  | Acute | Ref |  | Ref |  | Ref |  | Ref |  |
|  | **Medicine Availability** | |  |  |  |  |  |  |  |
|  | Available | 2.44 [1.38-4.31] | 0.002 | 2.62 [1.46-4.70] | **0.001** | 1.56 [0.93-2.61] | 0.089 | 1.67 [0.98-2.85] | 0.061 |
|  | Unavailable | Ref |  | Ref |  | Ref |  | Ref |  |
|  | **Knowledge of National Health Insurance Program** |  |  |  |  |  |  |  |  |
|  | Adequate | 1.97 [1.14-3.43] | 0.016 | 2.19 [1.22-3.92] | **0.009** | 1.38 [0.80-2.38] | 0.243 | 1.29 [0.72-2.33] | 0.381 |
|  | Inadequate |  |  |  |  |  |  |  |  |
