# Supplementary material for: Patient satisfaction among national health insurance enrollees in an accredited hospital of Kathmandu Valley: A cross-sectional, mixed methods study
Source: PLoS One. 2026 Mar 20;21(3):e0345353. doi: 10.1371/journal.pone.0345353 (PMC13004337; doi:10.1371/journal.pone.0345353)
Supplement: S1 Appendix — Includes all the items asked during the satisfaction survey and the key-informant interview guidelines. (DOCX) [file pone.0345353.s008.docx]

**S1 Appendix. Questionnaire and Interview Guidelines.** Includes all the items asked during the satisfaction survey and the key-informant interview guidelines.

# Survey Questionnaire

**Socio-demographic information**

| S.N. | Questions | Options | Code |
| --- | --- | --- | --- |
|  | Age | _____ (in completed years) | - |
|  | Sex | 1. Male  2. Female  3. Others (Please specify _____________)  4. Prefer not to say | 0  1  2  3 |
|  | Marital Status | 1. Never Married  2. Married  3. Living Together  4. Divorced  5. Separated  6. Widowed | 0  1  2  3  4  5 |
|  | Religion | 1. Hindu  2. Buddhist  3. Muslim  4. Kirat  5. Christian  6. No Religion  7. Others (Please specify ____________) | 0  1  2  3  4  5  6 |
|  | Ethnicity | 1. Brahmin  2. Chhetri  3. Dalit  4. Janajati  5. Madheshi  6. Muslim  7. Others (please specify_________) | 0  1  2  3  4  5  6 |
|  | Mother tongue/ Native Language | 1. Nepali  2. Maithili  3. Bhojpuri  4. Tharu  5. Newari  6. Tamang  7. Other (please specify ________) | 0  1  2  3  4  5  6 |
|  | Area of residence | 1. Urban  2. Rural | 0  1 |
|  | Education status (highest level of attainment regardless of completion) | 1. No education  2. Basic Education (1 – 8)  3. Secondary (9 – 12)  4. More than secondary (13 and above) | 0  1  2  3 |
|  | Income | ______________ (in NRP) | - |
|  | Occupation | 1. Agriculture  2. Business  3. Service (Government)  4. Service (Private)  5.Daily Labour  6. Student  7. Housewife  8. Unemployed  9. Others (Please specify_______________) | 0  1  2  3  4  5  6  7  8 |
|  | Family size | __________ | - |

**Individual factors**

| S.N. | Questions | Options | Code | Skip pattern |
| --- | --- | --- | --- | --- |
|  | Time to reach health facility (in min.) | ___________  (in minutes) | - |  |
|  | Self-reported health status | 1. Very Good  2. Good  3. Moderate  4. Bad  5. Very Bad | 0  1  2  3  4 |  |
|  | Type of illness | 1. Acute  2. Chronic | 0  1 |  |
|  | NHI premium affordability | 1. Affordable  2. Not affordable | 0  1 | If no, skip to question number 7 |
|  | Willingness to pay greater contribution for better insurance benefit package | 1. Yes  2. No | 0  1 |  |
|  | What is the maximum payment amount you are willing to pay for health insurance premium, given that it covers wider service areas and provides high quality of care and better ceiling? | __________  (in NRP) | - |  |
|  | Insurance type | 1. Subsidized  2. Non – subsidized | 0  1 |  |
|  | Years of enrollment | __________ months/years | - |  |
|  | Have you renewed your insurance every year since enrollment? | 1. No  2. Yes | 0  1 | If yes, skip to question number 24 |
|  | If not, what was the reason? | 1. Did not consider insurance necessary anymore  2. Renewal got overlooked  3. Could not contact enrollee assistant  4. Long waiting time  5. The quality of services was poor  6. Did not know the process of renewal  7. Others (please specify ___) | 0  1  2  3  4  5  6 |  |
|  | Do you believe that NHIP has benefits? | 1. No  2. Yes | 0  1 | If no, skip to question number 22 |
|  | What are the benefits of NHIP? | 1. Prevents unexpected healthcare expenditure  2. Help others who cannot afford their medical cost  3. Have timely care in time  4. Improve health service quality  5. All of the above | 0  1  2  3  4  5  6 |  |
|  | Are the prescribed drugs/medicines available in premises of hospital? | 1. Yes  2. Sometimes  3. No | - |  |

**Knowledge of NHIP**

| S.N. | Questions | Options | Code | Skip pattern |
| --- | --- | --- | --- | --- |
|  | Do you know about the provisions under national health insurance program? | 1. Yes  2. No | 0  1 | Skip Pattern |
|  | What are the provisions under national health insurance program? | 1. A family of five can get services through a premium amount of Rs. 3,500  2. Insurance programs runs on a voluntary mechanism  3. Insured can get services up to Rs. 1,00,000 and with every additional member, Rs. 700 is to be added to the premium amount  4. The insured member can get services from all government primary service point and get referred to government, private or community hospitals  5. All of the above | 1  2  3  4  5 | What are the provisions under national health insurance program? |
|  | Do you know of the services included in the benefit package? | 1. Yes  2. No | 0  1 | Do you know of the services included in the benefit package? |
|  | What services are included in the benefit package? (Multiple response) | 1. OPD services  2. Emergency services  3. Inpatient services  4. Diagnostic services (X-ray, ECG, Ultrasound, MRI, CT scan, blood test, etc.)  5. Listed medicines  6. Surgery, Appendix operation, Stone operation  7. Prescription glasses, hearing aid, white stick and Baisakhi at any rate | 0  1  2  3  4  5  6 | What services are included in the benefit package? (Multiple response) |
|  | Do you know of health services excluded by insurance benefit package? | 1. Yes  2. No | 0  1 | Do you know of health services excluded by insurance benefit package? |
|  | If yes, what are the services excluded by the insurance benefit package? (Multiple response) | 1. More than predetermined annual rate in prescription/vision glasses, white stick, hearing aid and crutches (Baisakhi)  2. Cosmetic surgery  3. Surgery for cleft lip and palate, serious forms of disability and burns  4. Dental treatment except for primary management of dental extraction, dental abscess or dental trauma  5. Surgery for burns | 0  1  2  3  4 | If yes, what are the services excluded by the insurance benefit package? (Multiple response) |
|  | If yes, what are the services excluded by the insurance benefit package? (Multiple response) | 1. More than predetermined annual rate in prescription/vision glasses, white stick, hearing aid and crutches (Baisakhi)  2. Cosmetic surgery  3. Surgery for cleft lip and palate, serious forms of disability and burns  4. Dental treatment except for primary management of dental extraction, dental abscess or dental trauma  5. Surgery for burns | 0  1  2  3  4 |  |

**Patient Satisfaction information**

On the following pages are some things people say about medical care. Please read each one carefully, keeping in mind the medical care you are receiving now. (If you have not received care recently, think about what you would expect if you needed care today.) We are interested in your feelings, good and bad, about the medical care you received.

How strongly do you AGREE or DISAGREE with each of the following statements?

| S.N. | Questions | Strongly Agree | Agree | Uncertain | Disagree | Strongly Disagree |
| --- | --- | --- | --- | --- | --- | --- |
| 1. | Doctors are good about explaining the reason for medical tests. | 1 | 2 | 3 | 4 | 5 |
| 2. | I think my doctor’s office has everything needed to provide complete medical care. | 1 | 2 | 3 | 4 | 5 |
| 3 | The medical care I have been receiving is just about perfect | 1 | 2 | 3 | 4 | 5 |
| 4. | Sometimes doctors make me wonder if their diagnosis is correct | 1 | 2 | 3 | 4 | 5 |
| 5. | I feel confident that I can get the medical care I need without being set back financially | 1 | 2 | 3 | 4 | 5 |
| 6. | When I go for medical care, they are careful to check everything when treating and examining me | 1 | 2 | 3 | 4 | 5 |
| 7 | I have to pay for more of my medical care than I can afford | 1 | 2 | 3 | 4 | 5 |
| 8. | I have easy access to the medical specialists I need | 1 | 2 | 3 | 4 | 5 |
| 9. | Where I get medical care, people have to wait too long for emergency treatment | 1 | 2 | 3 | 4 | 5 |
| 10. | Doctors act too businesslike and impersonal toward me | 1 | 2 | 3 | 4 | 5 |
| 11. | My doctors treat me in a very friendly and courteous manner | 1 | 2 | 3 | 4 | 5 |
| 12. | Those who provide my medical care sometimes hurry too much when they treat me | 1 | 2 | 3 | 4 | 5 |
| 13. | Doctors sometimes ignore what I tell them | 1 | 2 | 3 | 4 | 5 |
| 14. | I have some doubts about the ability of the doctors who treat me | 1 | 2 | 3 | 4 | 5 |
| 15. | Doctors usually ignore what I tell them | 1 | 2 | 3 | 4 | 5 |
| 16. | I find it hard to get an appointment for medical care I receive | 1 | 2 | 3 | 4 | 5 |
| 17. | I am dissatisfied with some things about the medical care I receive | 1 | 2 | 3 | 4 | 5 |
| 18. | I am able to get medical care whenever I need it | 1 | 2 | 3 | 4 | 5 |

# Qualitative Interview Guidelines

**Interview Guideline for Health Service Provider**

**Introductory Questions**

What is your work designation?

For how long have you worked in this institution?

What are your roles and responsibilities according to the nature of your position?

**Exploratory Questions**

1. Do you think patients seeking services in general are satisfied with health care service provided by the hospital?

*(Probe: How do you think the patient satisfaction is different among health insurance enrollees compared to that of general patients?)*

*(Probe: What do you think are the factors that make health insurance patients more or less likely to be satisfied with healthcare services? Probe: Medicine Availability, Knowledge of the health insurance program, accessibility and convenience)*

1. Do you think the equipment needed for the work is present adequately in the hospital department?

*(Probe: What do you think could be done better in terms of equipping the hospital department)*

1. Do you think health care providers are sufficiently available to provide healthcare services considering the outpatient flow of the hospital?

*(Probe: What additional services would be required to address the type of illnesses seen in insured patients?)*

1. How would you describe your interpersonal relations with patients?
2. How would you describe your interpersonal relations with your co-workers and hospital staff?
3. What are the opportunities for professional improvement at your current place of work?

*(Probe: Do you think they are adequate for your personal career development? If yes, why? If no, why?)*

1. What is the time available for accomplishment of your daily/usual tasks?
2. What is your salary?

*(Probe: Are you satisfied with the way reimbursement works under the national health insurance scheme? What could be done better?)*

1. What are your opportunities for continuous professional education?
2. How do you find the clarity of the instructions you receive regarding the expectations you need to meet at your workplace?
3. What were your experiences with the NHIP accreditation system?
4. Do you face any challenges with the claim reimbursement system?

**Exit/Closing questions**

1. What, in your opinion, could help you find greater motivation to conduct your duties and responsibilities?
2. What suggestions would you like to provide to the current provider payment mechanism or way of functioning of the national health insurance program?
3. Is there anything you would like to add in the end?
